# Antisense oligonucleotides reverse SPTLC1-related hereditary sensory neuropathy in a mouse model

**DOI:** 10.1093/brain/awaf403

**Published:** 2025-10-22

**Authors:** Jinhong Meng, Shunyi Ma, Museer A Lone, Hou Wang Lam, Qiang Zhang, Shuzhi Cheng, Shona Mackie, Emma Graham, Hanna Kedzior, Charalambos Demetriou, Nicole Ziak, Laura Andreoli, Simon Beggs, Stephanie Koch, Alex J Clark, David L Bennett, Thorsten Hornemann, Francesco Muntoni, Mary M Reilly, Haiyan Zhou

**Affiliations:** Genetics and Genomic Medicine Department, Great Ormond Street Institute of Child Health, University College London, London WC1N 1EH, UK; NIHR Great Ormond Street Hospital Biomedical Research Centre, UCL Great Ormond Street Institute of Child Health, London WC1N 1EH, UK; Genetics and Genomic Medicine Department, Great Ormond Street Institute of Child Health, University College London, London WC1N 1EH, UK; Institute for Clinical Chemistry, University Hospital and University of Zürich, 8006 Zürich, Switzerland; Genetics and Genomic Medicine Department, Great Ormond Street Institute of Child Health, University College London, London WC1N 1EH, UK; Genetics and Genomic Medicine Department, Great Ormond Street Institute of Child Health, University College London, London WC1N 1EH, UK; Genetics and Genomic Medicine Department, Great Ormond Street Institute of Child Health, University College London, London WC1N 1EH, UK; Genetics and Genomic Medicine Department, Great Ormond Street Institute of Child Health, University College London, London WC1N 1EH, UK; Genetics and Genomic Medicine Department, Great Ormond Street Institute of Child Health, University College London, London WC1N 1EH, UK; Genetics and Genomic Medicine Department, Great Ormond Street Institute of Child Health, University College London, London WC1N 1EH, UK; Genetics and Genomic Medicine Department, Great Ormond Street Institute of Child Health, University College London, London WC1N 1EH, UK; The Dubowitz Neuromuscular Centre, Great Ormond Street Institute of Child Health, University College London, London WC1N 1EH, UK; Institute for Clinical Chemistry, University Hospital and University of Zürich, 8006 Zürich, Switzerland; Department of Neuroscience, Physiology and Pharmacology, University College London, London WC1E 6BT, UK; Department of Neuroscience, Physiology and Pharmacology, University College London, London WC1E 6BT, UK; Department of Neuroscience, Physiology and Pharmacology, University College London, London WC1E 6BT, UK; Centre of Neuroscience, Surgery and Trauma, Blizard Institute, Barts and the London School of Medicine and Dentistry, London E1 2AT, UK; Neural Injury Group, Nuffield Department of Clinical Neuroscience, John Radcliffe Hospital, University of Oxford, Oxford OX3 9DU, UK; Institute for Clinical Chemistry, University Hospital and University of Zürich, 8006 Zürich, Switzerland; NIHR Great Ormond Street Hospital Biomedical Research Centre, UCL Great Ormond Street Institute of Child Health, London WC1N 1EH, UK; The Dubowitz Neuromuscular Centre, Great Ormond Street Institute of Child Health, University College London, London WC1N 1EH, UK; Neurodegenerative Disease Department, UCL Queen Square Institute of Neurology, London WC1N 3BG, UK; Department of Neuromuscular Diseases, UCL Queen Square Institute of Neurology, London WC1N 3BG, UK; Genetics and Genomic Medicine Department, Great Ormond Street Institute of Child Health, University College London, London WC1N 1EH, UK; NIHR Great Ormond Street Hospital Biomedical Research Centre, UCL Great Ormond Street Institute of Child Health, London WC1N 1EH, UK

**Keywords:** neurodegenerative disorder, antisense oligonucleotide (ASO), allele specific silencing, peripheral neuropathy, mouse model, biodistribution

## Abstract

Hereditary sensory neuropathy type IA (HSN1A) is a rare neurodegenerative condition caused by dominant mutations in the *Serine Palmitoyl Transferase Long Chain base subunit 1* (*SPTLC1*) gene. There is no treatment available.

Allele-specific silencing by antisense oligonucleotides (ASOs) to preferentially silence the mutant transcripts has shown therapeutic promise for dominant gain-of-function genetic disorders. In this study, we validated an allele-specific ASO therapy to selectively silence mutant SPTLC1 (p.S331F) in a disease mouse model carrying a heterozygous p.S331F mutation (S331F mice).

Gapmer ASOs, targeting the S331F variant in either 2′-*O*-methyl (2′-OMe), locked nucleic acid (LNA) or 2′-*O*-methoxy ethyl (MOE) chemistries, were first studied in cultured mouse skin fibroblasts. The candidate ASOs in LNA or MOE were further evaluated *in vivo*.

Single subcutaneous injection of ASOs into neonatal or adult S331F mice achieved over 90% mutant transcript silencing in liver and dorsal root ganglia (DRG). Weekly subcutaneous injections of LNA-ASOs, either unconjugated or conjugated with *N*-acetylgalactosamine (GalNAc), into S331F mice showed GalNAc-LNA-ASOs to be more efficient than unconjugated LNA-ASOs at reducing mutant transcripts in liver, DRG and sciatic nerve, without affecting wild-type transcripts. GalNAc-LNA-ASOs also resulted in significantly reduced blood levels of 1-deoxysphingoid bases (1-deoxySL), neurotoxic metabolites used as biomarkers in HSN1A patients.

Transcriptomic studies in DRG demonstrated mitochondrial pathway involvement in the pathological changes observed in S331F mice. Quantitative RT-PCR confirmed the differentially expressed genes between S331F and wild-type mice. Furthermore, these aberrantly expressed genes in S331F mice were reversed by GalNAc-LNA-ASO treatment. Our data provide necessary *in vivo* evidence as proof of concept for ASO-mediated mutant-allele-specific silencing as a therapeutic approach for SPTLC1-related HSN1.

## Teaser

Meng *et al.* have investigated the use of antisense oligonucleotides as a therapeutic strategy for sensory neuropathy resulting from *SPTLC1* mutations. In a mouse model, allele-specific ASOs selectively silenced the mutant transcript, providing proof of concept for this targeted approach.

## Introduction

**Figure 1:**
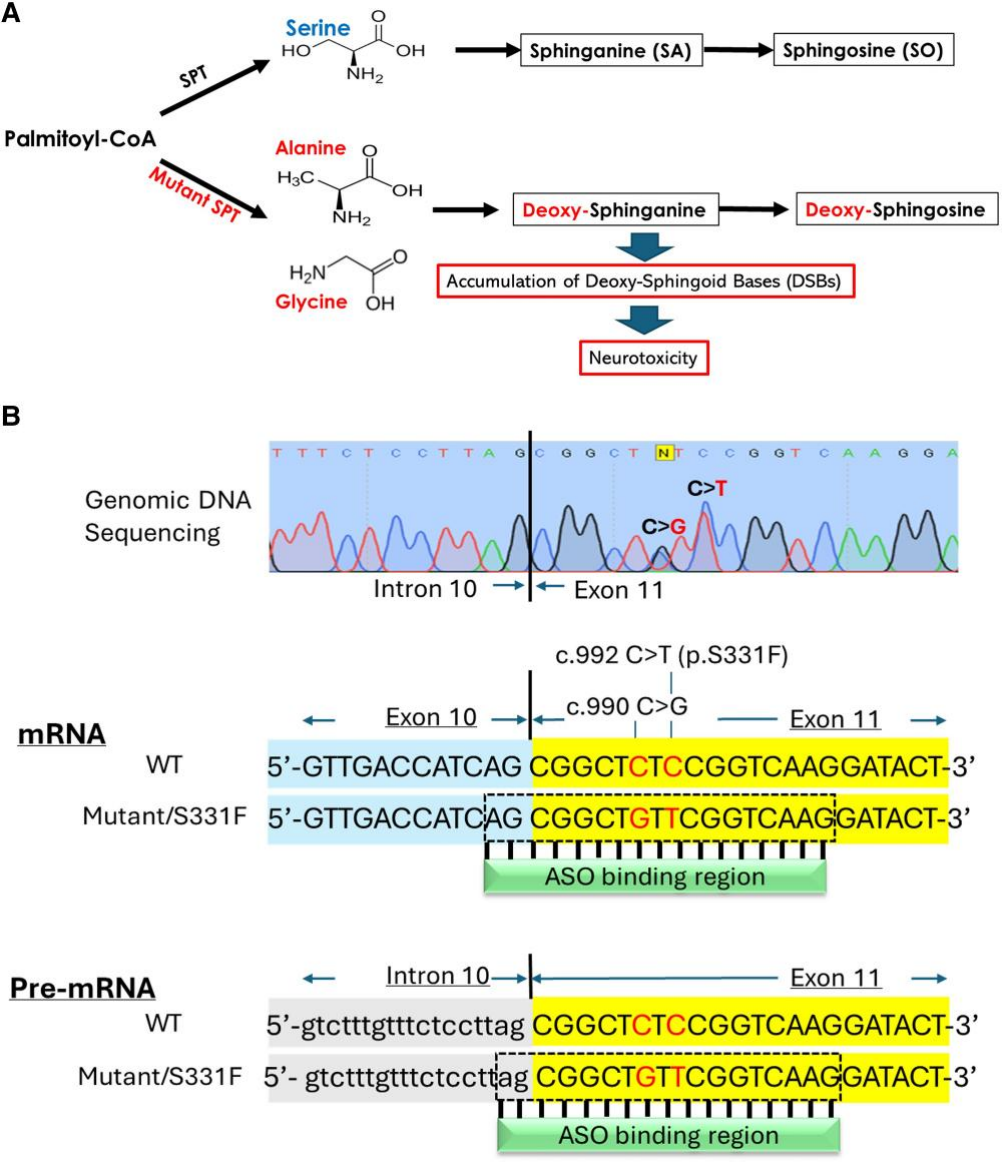
**SPTLC1-related sphingolipid metabolic pathways and the genetic defect in the S331F mouse model**. (**A**) SPT catalyses the first step in the synthesis of sphingolipids by conjugating palmitoyl-CoA and L-serine. Mutations in *SPTLC1* reduce the affinity of the enzyme for L-serine and increase its affinity for alanine and glycine, thereby leading to the formation and accumulation of neurotoxic 1-deoxy-sphingoid bases (DSBs). (**B**) The S331F mouse model carries two heterozygous variants, c.990 C>G and c.992 C>T (p.S331F) in exon 11 of the mouse *Sptlc1* gene, generated by CRISPR/Cas9 genome editing. ASOs were designed to target the indicated ASO binding region on the mutant allele at both mRNA and pre-mRNA levels. ASO = antisense oligonucleotide; WT = wild-type.

Hereditary sensory neuropathy type I (HSN1) is a neurodegenerative disorder, affecting 1 in 500 000 individuals in the general population.^1^ It is characterized by prominent sensory loss, neuropathic pain, and various degrees of limb weakness in advanced cases.^2^ Loss of sensation can lead to painless injuries, resulting in slow wound healing, osteomyelitis and subsequent distal amputations. HSN1A is caused by mutations in the *Serine Palmitoyl Transferase Long Chain base subunit 1* (*SPTLC1*) gene, which encodes a subunit of serine palmitoyl transferase (SPT).^3^ SPT catalyses the initial step in sphingolipid biosynthesis by conjugating palmitoyl-CoA with L-serine. Mutations in *SPTLC1* cause a shift in the substrate specificity of SPT, from serine to alanine and glycine, leading to the production of 1-deoxy-sphingolipids (1-deoxySLs) (Fig. 1A), which are toxic to neurons.^4-6^ As 1-deoxySLs are resulted from a gain-of-function effect, secondary to missense mutations in the *SPTLC1* gene, and the fact that haploinsufficiency of SPTLC1 is not pathogenic,^4,7,8^ selective silencing of the mutant transcripts while retaining the wild-type (WT) allele may eliminate 1-deoxySL formation. Such an approach is particularly relevant, given that SPTLC1 is essential for the synthesis of canonical sphingolipids, which play key roles in cell adhesion, intercellular signalling and membrane dynamics, and that homozygous loss-of-function in Sptlc1 is embryonically lethal in mice.^8,9^

RNA-targeted therapy using antisense oligonucleotides (ASOs) offers great potential for neurodegenerative disorders. Gene-silencing ASOs have been used successfully in treating conditions caused by gain-of-function mutations, such as innotersen for TTR-related amyloidosis,^10^ volanesorsen for familial chylomicronaemia syndrome^11^ and tofersen for amyotrophic lateral sclerosis (ALS).^12^ While general downregulation of a protein, especially an enzyme, could be detrimental, a specific lowering of the mutant protein would be of benefit in some conditions. Selective mutant-allele downregulation has been developed in Huntington’s disease as an alternative to improve on previous clinical trials using a general gene-silencing approach.^13^ Allele-specific silencing ASOs have also been investigated in other dominant gain-of-function conditions, such as COL6-related muscular dystrophy.^14,15^ A small interfering RNA (siRNA) approach was tested in fibroblasts cultured from patients with childhood ALS caused by dominant *SPTLC1* mutations, a severe allelic condition to HSN1A, which silenced mutant transcripts and normalized the elevated levels of canonical SPT.^16^

Here, we investigate an allele-specific ASO approach in an existing Sptlc1 mouse model harbouring a heterozygous S331F mutation in *Sptlc1*. We describe *in vivo* proof-of-concept studies of developing ASOs targeting the S331F mutation in mice. ASOs in 2′-*O*-methyl (2′-OMe), 2′-*O*-methyloxyethyl (2′-MOE) and locked nucleic acids (LNA) were tested in mouse skin fibroblasts. The lead ASOs in LNA or MOE, as well as LNA conjugated with *N*-acetylgalactosamine (GalNAc), were further evaluated in S331F mice. Our data show a specific suppression of the mutant *Sptlc1* mRNA *in vivo*, without affecting the WT *Sptlc1* transcripts. Repeated systemic ASO treatment significantly reduced 1-deoxySL in blood, confirming that plasma 1-deoxySL levels are a sensitive therapeutic biomarker. Using next-generation mRNA sequencing, we also identify mitochondrial pathway involvement in S331F mice, which appears to be modulated by effective ASO treatment. Our results demonstrate the feasibility of an allele-specific ASO approach in treating SPTLC1-related HSN1 (SPTLC1-HSN1).

## Materials and methods

### Study design

Studies were designed to investigate ASOs for silencing mutant transcripts in S331F mice and to assess their effects on plasma levels of 1-deoxySL and on the transcriptomes in dorsal root ganglia (DRG) and liver. ASOs designed with different chemical modifications were tested in skin fibroblasts derived from S331F mice. The specific silencing effect on S331F transcripts was measured by allele-specific real-time PCR (qRT-PCR). The lead ASOs in MOE or LNA chemistries were then tested in neonatal or young-adult S331F mice treated with a single subcutaneous injection. Liver, DRG and sciatic nerve samples were assessed by qRT-PCR at 7 days post-injection. The lead ASOs in MOE, LNA and GalNAc-conjugated LNA were further studied in adult S331F mice treated with weekly subcutaneous injections for 8 weeks, followed by: qRT-PCR of mRNA expression in liver, DRG and sciatic nerves; mass spectrometry measurement of plasma 1-deoxySL levels; and transcriptomic studies using next-generation mRNA sequencing in DRG. Age-matched saline-treated S331F mice were used as controls. The number of mice required for each experiment was determined through pilot studies and justified in accordance with the 3Rs principle. All experimental and control mice were allocated randomly, and all studies were conducted in a double-blind manner.

### ASOs

ASOs for *in vitro* studies were synthesized by Eurogentec Ltd. ASOs for *in vivo* studies were synthesized by Microsynth Ltd. GalNAc-conjugated ASOs were synthesized by AxoLabs GmbH.

### Mouse procedures

The S331F mice (C57BL/6NTac-Sptlc1^em1H^/H, stock code: SPTLC1-S331F-EM1-B6N) were obtained from the Mary Lyon Centre at MRC Harwell.^17^ Mice heterozygous for S331F mutation (*S331F^+/-^*) were crossed with WT C57BL/6N mice. Subcutaneous injections, blood and tissue collection were carried out in the Biological Services Unit, University College London, in accordance with the Animals (Scientific Procedures) Act 1986. Experiments were performed under Home Office licence number PP2611161.

### Mouse fibroblast culture and antisense oligonucleotide treatment

Skin fibroblasts were cultured from S331F mice. For Lipofectamine 2000 transfection, cells were seeded into 24-well plates at a density of 5 × 10^4^ cells/well, giving 80% confluence on the next day. ASOs were complexed with Lipofectamine 2000 (Thermo Fisher) in Opti-MEM, according to the manufacturer’s instructions and incubated with cells for 24 h before RNA extraction. For gymnotic treatment, fibroblasts were seeded at a density of 2 × 10^4^ cells/well. The next day, ASOs were added to growth medium and cultured for 6 days before RNA extraction.

### Allele-specific quantitative real-time reverse transcription PCR

Total RNA from cultured cells or mouse tissues was extracted using the Qiagen RNeasy Mini kit. Quantification of WT or S331F *Sptlc1* mRNA was performed using iTaq Universal One-Step RT-qPCR Kit (Bio-Rad), with mouse *Hprt* as the housekeeping gene. Primer sequences are provided in Supplementary Table 1.

### SplintR PCR

Concentrations of ASOs in mouse tissues were measured by SplintR PCR according to protocol described previously.^18^ Mouse tissues were lysed in RIPA buffer. The supernatant of tissue lysate was hybridized and ligated with probes using a SplintR ligase kit (New England Biolabs, M0375S), following manufacturer’s instructions. After ligation, qRT-PCR was performed using a PrimeTime™ Gene Expression Master Mix kit (Integrated DNA Technologies, 1055772) with primers and probes specifically designed to recognize the target ASOs (MOE-ASO1 and LNA-ASO1). The sequences of probes and primers are provided in Supplementary Table 5.

### Mass spectrometry analysis

Plasma levels of 1-deoxySL were measured by multiple reaction monitoring-based liquid chromatography-mass spectrometry (LC-MRM-MS) as previously described.^19^ Briefly, lipid was extracted from a 50 µl plasma sample by adding 50 µl of deionized water, followed by 500 µl of the lipid extraction solvent (100% methanol containing D7-C18SO and D7-C18SA, 200 pmol per sample). A chemical hydrolysis step was added to the extracted lipids prior to analysis. Long-chain bases of sphingolipids extracted from lipid hydrolysates were quantified by MS using D7-C18SO and D7-C18SA as the internal standards. Mass spectra were subsequently collected in MRM positive ion mode. 1-deoxySO and 1-deoxySA were detected in the single dehydration product generated at the ion source using a declustering potential of 160 V. The sum of 1-deoxySO and 1-deoxySA concentrations was quantified as the level of 1-deoxySL.

### Transcriptomics and RNA sequencing analysis

DRGs from all spinal levels were dissected from 3-month old male WT and S331F mice. Total RNA was extracted and quality controlled using an Agilent Bioanalyser 2100 TapeStation system (UCL Genomics). Next-generation RNA sequencing was performed by Novogene (UK). FPKM (the expected number of Fragments Per Kilobase of transcript sequence per Millions base pairs sequenced), representing the abundance of transcripts (count of sequencing) that mapped to the genome or exon, was used to estimate the level of gene expression. For high-confidence expression, FPKM ≥ 10 was used to filter out low-abundance transcripts. Non-coding mouse transcripts were excluded from the subsequent gene analysis. Differentially expressed genes (DEGs), analysed by the DESeq2 method, were determined when the event had sufficient read coverage (coverage ≥20), log_2_(FoldChange) ≥ 0, *P* ≤ 0.01 and false discovery rate (FDR) ≤ 0.05. Enrichment studies were performed using gene ontology (GO), Kyoto Encyclopedia of Genes and Genomes (KEGG) and Reactome database enrichment analyses of biological functions or pathways significantly associated with the DEGs. GO terms, KEGG pathways and Reactome pathway enrichment with an adjusted *P-*value (*P*adj) < 0.05 were deemed as significant enrichment.

### Statistics

Comparisons between two groups were performed using unpaired Student’s *t*-test (normal data) or Mann–Whitney U-test (non-normal data), as appropriate. For comparison of three or more groups, one-way ANOVA followed by Tukey’s test was used for normal data, or Kruskal-Wallis test followed by Dunn’s test for non-normal data. Data were analysed using two-way ANOVA and Sidak’s multiple comparisons test for comparisons involving more than two groups across two independent factors [e.g. factors: (WT versus mutant transcripts) × (saline versus ASO treatment)]. Assumptions were verified via normality tests. Significance was set at *P* < 0.05. Results presented in this study are displayed as mean ± standard error of the mean. GraphPad Prism 10.0 software was used for statistical analysis and graph design.

## Results

### ASOs selectively silence mutant transcripts in S331F mouse skin fibroblasts

S331F mice carry two heterozygous variants, c.990 C>G (a synonymous base change created during CRISPR/Cas9 genome editing when generating the c.992 C>T mutation) and c.992 C>T (the missense S331F variant) in *cis* in exon 11 of the mouse *Sptlc1* gene (Fig. 1B). Nine gapmer ASOs were designed with 2′-OMe, MOE or LNA chemical modifications. The two nucleotides complementary to c.990G and c.992T were placed in the central phosphorothioate-DNA sequence (Table 1).

**Table 1:** Antisense oligonucleotides tested in this study

ID	Chemistry	ASO sequence	Length (Mer)
OMe-ASO1	2′-OMe	[UUGA]C*C*G*A*A*C*A*G*C*[CGCU]	17
OMe-ASO2	2′-OMe	[UUGA]C*C*G*A*A*C*A*G*C*C*[GCU]	17
OMe-ASO3	2′-OMe	[CUUG]A*C*C*G*A*A*C*A*G*C*[CGCU]	18
MOE-ASO1	MOE	<UUGA>C*C*G*A*A*C*A*G*C*<CGCU>	17
MOE-ASO2	MOE	<UUGA>C*C*G*A*A*C*A*G*C*C*<GCU>	17
MOE-ASO3	MOE	<CUUG>A*C*C*G*A*A*C*A*G*C*<CGCU>	18
LNA-ASO1	LNA	{UUGA}C*C*G*A*A*C*A*G*C*{CGCU}	17
LNA-ASO2	LNA	{UUGA}C*C*G*A*A*C*A*G*C*C*{GCU}	17
LNA-ASO3	LNA	{CUUG}A*C*C*G*A*A*C*A*G*C*{CGCU}	18

Phosphorothioate (PS)-modified DNA nucleotides are indicated with an asterisk; RNA nucleotides modified with 2′-*O*-methyl (2′-OMe) chemistry are included in square brackets; RNA nucleotides modified with 2′-*O*-methoxy ethyl (MOE) chemistry are included in angle brackets; RNA nucleotides modified with locked nucleic acid (LNA) chemistry are included in curly brackets. Nucleotides complementary to c.990G and c.992T are underlined. ASO = antisense oligonucleotide.

**Figure 2:**
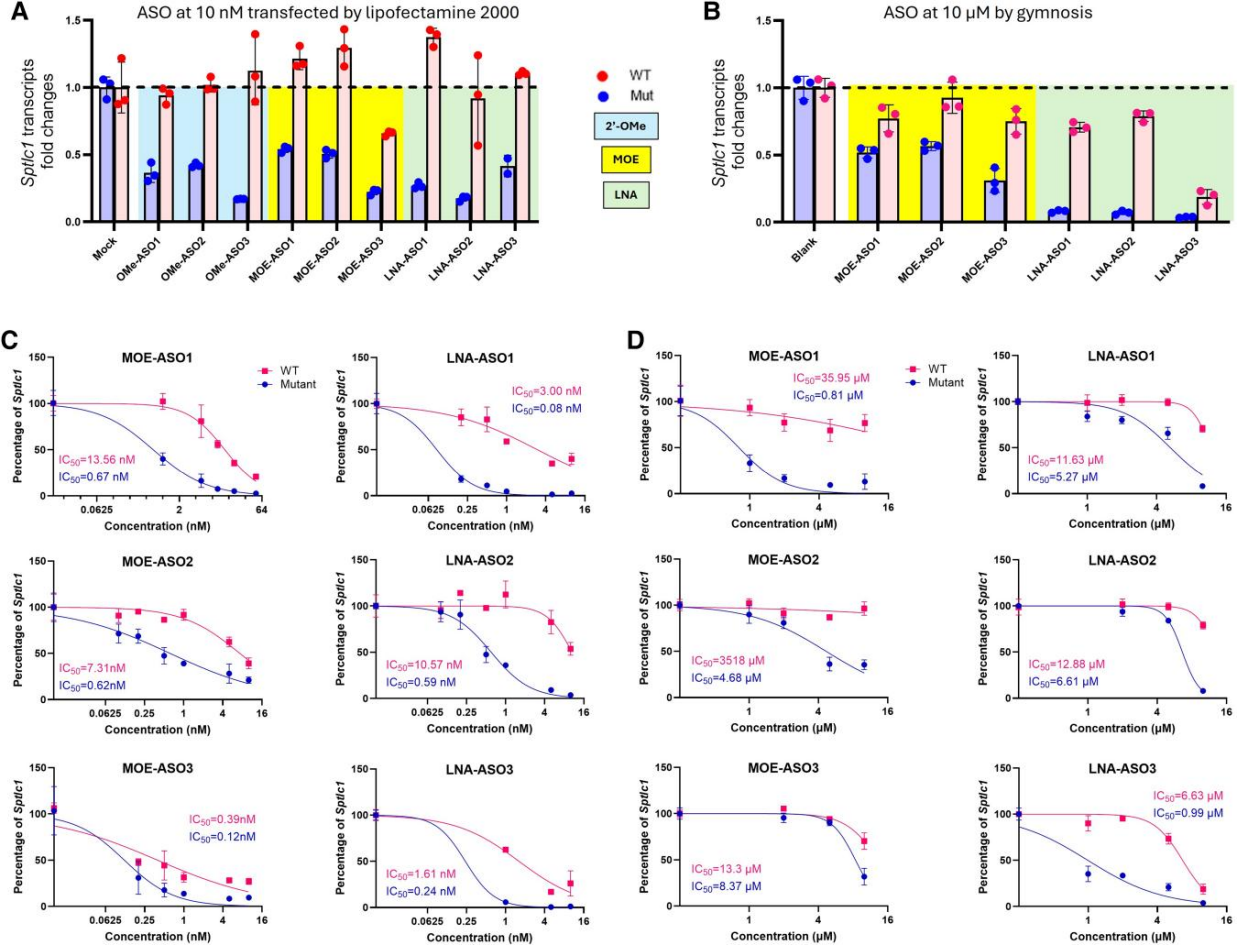
**ASOs silence the mutant transcripts in skin fibroblasts cultured from S331F mice**. Nine ASOs were designed in 2’-OMe, MOE or LNA chemistry, and tested in cultured skin fibroblasts from mice either at 10 nM by (**A**) lipofectamine 2000 transfection or (**B**) at 10 µM by gymnosis. Wild-type (WT) and mutant (Mut) transcripts were quantified by allele-specific qRT-PCR, normalized to the house keeping gene, *Hprt1*. The levels of corresponding transcripts in mock or blank treated groups were used as controls. Dose-response studies of MOE and LNA-ASOs were performed in mouse fibroblasts treated by (**C**) lipofectamine 2000 transfection in a concentration range of 0 to 64 nM or (**D**) gymnosis in a concentration range of 0 to 16 µM. The IC50 values of ASOs on the mutant (in red) or WT (in blue) transcripts were measured and indicated. 2′-OMe = 2′-*O*-methyl; ASO = antisense oligonucleotide; IC50 = median inhibition concentration; LNA = locked nucleic acid; MOE = 2′-*O*-methoxy ethyl.

Skin fibroblasts cultured from S331F mice were treated with ASOs at 10 nM for 24 h with Lipofectamine 2000, as the first round of ASO screening. The mutant and WT transcripts were measured by allele-specific qRT-PCR (primers listed in Supplementary Table 1). All nine ASOs exhibited significant effects in distinguishing between mutant and WT transcripts by presenting more preferential silencing effects on mutant than WT transcripts (Fig. 2A). We next performed gymnosis studies on MOE and LNA-ASOs, considering their potential and satisfactory safety profile in clinical application. Fibroblasts were treated with ASOs at 10 µM for 6 days, in the absence of transfection reagent. Except for LNA-ASO3, which downregulated both WT and mutant transcripts, all other ASOs showed efficient and preferential silencing on the mutant transcripts (Fig. 2B)

We next conducted dose-response studies of MOE- and LNA-ASOs (Fig. 2C and D). Mouse fibroblasts were treated with ASOs at a series of concentrations ranging from 0 to 64 nM under transfection with Lipofectamine 2000 (Fig. 2C). Median inhibition concentration (IC50) values of ASOs on either mutant (IC50 Mut) or WT (IC50 WT) transcripts were used as parameters to indicate silencing efficiency and specificity. The ratio of IC50 WT to IC50 Mut was also used to identify the lead compounds showing the highest specificity (Supplementary Table 1). The higher the IC50 WT/Mut, the more specific the ASO is for mutant over WT transcripts. LNA-ASO1 was determined to be the most potent candidate, with IC50 Mut = 0.08 nM, IC50 WT = 3.00 nM and IC50 WT/Mut = 37.5. MOE-ASO1, which shares the same nucleotide sequence as LNA-ASO1, also showed superior specificity in silencing mutant transcripts, with IC50 Mut = 0.67 nM, IC50 WT = 13.56 nM and IC50 WT/Mut = 20.24. LNA-ASO3 and MOE-ASO3 were also efficient at silencing mutant transcripts; however, they showed low specificity, silencing WT transcripts at the same time (Fig. 2C and Supplementary Table 2).

This was followed by dose-response gymnosis studies with ASOs at a concentration range of 0–16 µM (Fig. 2D). MOE-ASO1 showed the lowest IC50 Mut = 0.81 µM, followed by LNA-ASO3, with IC50 Mut = 0.99 µM. However, LNA-ASO3 also exhibited a strong silencing effect on the WT allele (IC50 WT = 6.63 nM), suggesting low discrimination between the two alleles (Fig. 2D and Supplementary Table 2).

Based on data from both lipofectamine transfection and gymnosis studies, we selected LNA-ASO1, LNA-ASO3 and MOE-ASO1 for further *in vivo* validation in S331F mice.

### LNA-ASO1 selectively silences mutant transcripts in S331F mice

Considering the significant silencing effect of LNA-ASOs observed *in vitro*, our initial *in vivo* studies were focused on LNA-ASO1 and LNA-ASO3. To determine the lead ASO, we started with single-dose studies in neonatal and adult mice.

**Figure 3:**
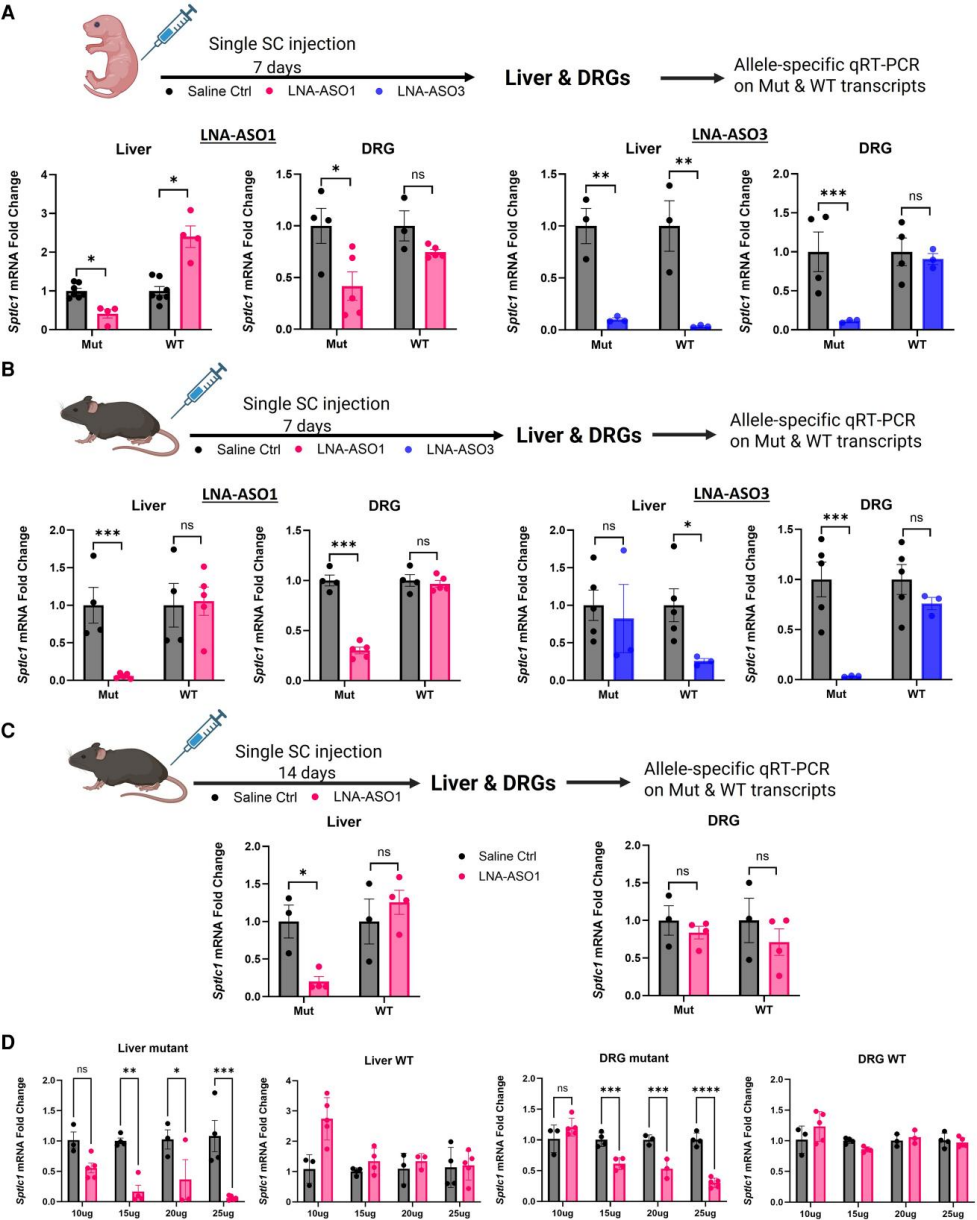
**LNA-ASOs selectively silence the mutant transcripts *in vivo* in the S331F mice**. (**A**) Newborn S331F mice at postnatal Day 3 and (**B**) young adult mice at 6 weeks old received a single subcutaneous (SC) injection of LNA-ASO1 or LNA-ASO3 at 25 µg/g. Liver and DRG were collected 7 days after the injection. Wild-type (WT) and mutant (Mut) *Sptlc1* mRNA levels in ASO-treated mice were measured by allele-specific qRT-PCR and normalized to saline control mice. (**C**) The duration of the silencing effect in the liver and DRG in adult mice after a single SC injection of LNA-ASO1 at 25 µg/g was measured at 14 days after the injection by allele-specific qRT-PCR and normalized to saline control mice. (**D**) Dose-response studies were performed in 6-week-old mice treated with a single subcutaneous injection of LNA-ASO1 at 10, 15, 20 and 25 µg/g. The allele-specific silencing was measured by qRT-PCR in the liver and DRG 7 days after the injection. Two-way ANOVA and Sidak’s multiple comparisons test were performed for statistical significance. *n* = 3–6 mice/group. *P < 0.05, **P < 0.01, ***P < 0.001, ****P < 0.0001. ASO = antisense oligonucleotide; DRG = dorsal root ganglia; LNA = locked nucleic acid. Created in BioRender. Zhou, H. (2025) https://BioRender.com/8css706.

Newborn S331F mice at postnatal Day 3 (PND3) received a single subcutaneous injection of LNA-ASO1 or LNA-ASO3 at 25 µg/g. Liver and DRG were collected at 7 days after the injection for subsequent allele-specific qRT-PCR analysis. In mice treated with LNA-ASO1, specific suppression of the mutant allele was detected, resulting in a 58% reduction in both the liver and DRG, compared to saline-treated age-matched S331F control mice. There was no significant silencing on WT transcripts in either liver or DRG (Fig. 3A). However, in mice treated with LNA-ASO3, mutant allele-specific silencing was only detected in the DRG, with an 88% reduction in mutant transcripts and no effect on WT transcripts. Significant reductions in both mutant and WT transcripts were detected in the liver, at 90% and 96%, respectively (Fig. 3A).

The distinct allele-specific silencing effect of LNA-ASO1 was also confirmed in 6-week-old adult S331F mice. A single subcutaneous injection of LNA-ASO1 at 25 µg/g significantly reduced mutant transcripts by 94% in liver and 70% in DRG (Fig. 3B). No silencing effect was detected on WT transcripts in either liver or DRG. In contrast, LNA-ASO3 showed no significant silencing effect on mutant transcripts (18%) in liver but a significant silencing effect on WT transcripts (74%). In DRG, 96% mutant allele-specific silencing was detected when compared to saline-treated controls (Fig. 3B). The data from the single dose studies suggested LNA-ASO1 as the lead ASO in achieving the desired mutant-allele specific silencing in both neonatal and adult mice. LNA-ASO1 was therefore selected as the lead compound for the subsequent *in vivo* studies.

Next, we assessed the duration of the silencing effect following a single subcutaneous injection. Six-week-old adult S331F mice were injected with LNA-ASO1 at 25 µg/g. The liver and DRG were harvested at 14 days post-injection, followed by allele-specific qRT-PCR analysis. The results showed that while there was still around 80% mutant allele silencing in liver, no silencing effect in DRG was detectable at 2 weeks after a single injection (Fig. 3C). These data suggested that repeated administration of LNA-ASO1 on a weekly basis may be necessary to achieve a long-term, sustainable therapeutic effect.

To determine the optimal dose of LNA-ASO1 for repeated treatments, we conducted a dose-response study at 10, 15, 20 and 25 µg/g and measured the allele-specific silencing effect by qRT-PCR in liver and DRG at 7 days after a single injection. In liver, significant mutant allele silencing was detected at all four doses, with a striking effect at the 15 µg/g dose, resulting in 83% silencing. In DRG, mutant transcripts remained unaffected at the 10 µg/g dose. Silencing became evident from 15 µg/g, with 39% silencing (Fig. 3D). No effect was detected in the WT allele in either liver or DRG. Our results present a clear dose-dependent effect of LNA-ASOs on mutant-allele-specific silencing *in vivo*. The 15 µg/g dose was carried forward for the following repeated LNA-ASO1 treatment.

### GalNAc conjugated LNA-ASO1 exerts improved allele-specific silencing in S331F mice

The liver is a central organ in lipid metabolism, including the biosynthesis of sphingolipids. To increase the bio-engagement of ASOs in hepatocytes, we conjugated LNA-ASO1 with GalNAc, the ligand to the asialoglycoprotein receptor (ASGPR), which is highly expressed in hepatocytes. Conjugation of ASOs to GalNAc can enhance their hepatocyte uptake by 20–30-fold.^20^

**Figure 4:**
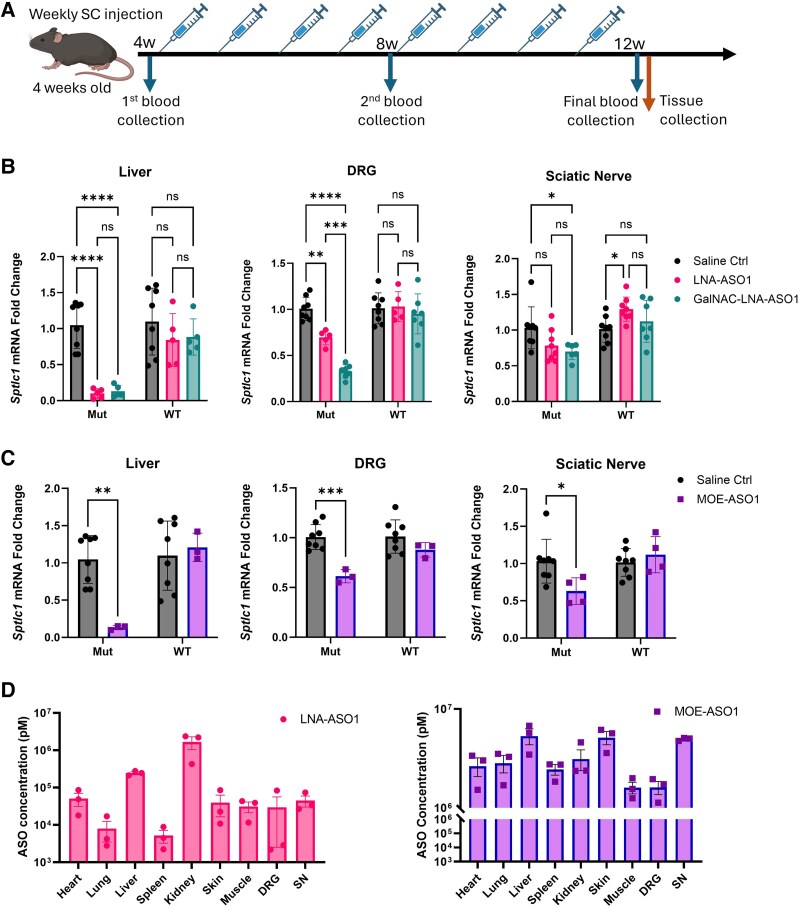
**Repeated weekly ASO treatments selectively silence mutant transcripts in S331F mice *in vivo***. (**A**) The timeline of repeated ASO treatments. Four-week-old mice received weekly subcutaneous injections of LNA-ASO1, GalNAc-LNA-ASO1 or MOE-ASO1 for 8 weeks. Liver, DRG and sciatic nerves were collected for allele-specific qRT-PCR 1 week after the eighth treatment. Blood samples were collected at baseline (4 weeks old), just before the first injection, and after 4 and 8 weeks of treatment. (**B**) The silencing effects of LNA-ASO1 and GalNAc-LNA-ASO1 in the liver, DRG and sciatic nerves after 8 weeks of treatment were measured by allele-specific qRT-PCR. The mutant (Mut) and wild-type (WT) *Sptlc1* mRNA levels were compared between LNA-ASO1 and GalNAc-LNA-ASO1 and normalized to saline controls. (**C**) The silencing effects of MOE-ASO1 in the liver, DRG and sciatic nerves after 8 weeks of treatment were measured by allele-specific qRT-PCR. The Mut and WT *Sptlc1* mRNA levels in the ASO-treated group were normalized to saline controls. (**D**) The tissue concentrations of ASOs in peripheral organs were measured by SplintR PCR. Two-way ANOVA and Sidak’s multiple comparisons test were performed for statistical significance. *n* = 3–8 mice/group. *P < 0.05, **P < 0.01, ***P < 0.001, ****P < 0.0001. ASO = antisense oligonucleotide; DRG = dorsal root ganglia; LNA = locked nucleic acid; MOE = 2′-O-methoxy ethyl. Created in BioRender. Zhou, H. (2025) https://BioRender.com/j3joxe5.

Considering first symptoms appear between the second and third decades of life in most patients with SPTLC1-associated HSN1, we decided to conduct the repeated injection experiment in young-adult mice. Four-week-old S331F mice were treated with weekly subcutaneous injections of either LNA-ASO1 at 15 µg/g or GalNAc-LNA-ASO1 at 4 µg/g, for a total of eight injections (Fig. 4A). One week after the last injection, liver, DRGs and sciatic nerves were harvested for allele-specific qRT-PCR analysis.

In liver, a significant downregulation of mutant transcripts was detected in both LNA-ASO1 and its GalNAc conjugates, at 90% (*P* < 0.0001) and 88% (*P* < 0.0001), respectively (Fig. 4B). In the DRG, 31% downregulation of mutant transcripts was detected in LNA-ASO1-treated mice (*P* = 0.0023), compared to 67% downregulation in the GalNAc-LNA-ASO1-treated mice (*P* < 0.0001) (Fig. 4B). A significant difference was detected between the LNA-ASO1 and GalNAc-LNA-ASO1 groups (*P* = 0.0005). In sciatic nerves, 24% mutant transcript downregulation was detected in LNA-ASO1-treated mice, which was further improved to 32% downregulation in GalNAc-LNA-ASO1-treated mice (*P* = 0.0252). No significant effects on WT transcripts were detected in liver, DRG or sciatic nerves from LNA-ASO1- or GalNAc-LNA-ASO1-treated mice (Fig. 4B).

### Repeated MOE-ASO treatment specifically downregulates mutant transcripts in S331F mice

Our *in vitro* studies indicated MOE-ASO1 as the second most efficient ASO after LNA-ASO1 in specifically silencing mutant transcripts (Fig. 2C). However, in the following *in vivo* studies in either newborn or adult S331F mice, a single subcutaneous injection of MOE-ASO1 at 50 µg/g did not show any silencing effects on mutant transcripts (Supplementary Fig. 1). Nevertheless, considering the wide and promising application of MOE chemistry in ASO clinical translations, we continued the studies of repeated MOE-ASO1 treatment and its long-term efficacy in mice. Four-week-old S331F mice received weekly subcutaneous injections of MOE-ASO1 at 50 µg/g for 8 weeks. One week after the last injection, liver, DRG and sciatic nerves were harvested for allele-specific qRT-PCR analysis. Significant mutant-allele-specific silencing was detected in all three organs, with 84% downregulation in liver (*P* = 0.001), 39% in DRG (*P* = 0.0007) and 39% in sciatic nerves (*P* = 0.02) (Fig. 4C). No silencing effects on WT transcripts were detected in any of the organs.

### ASOs reach high concentrations in peripheral organs after 8 weeks of treatment

SPTLC1 is ubiquitously expressed, and sphingolipid biosynthesis is essential in all organs. To understand the *in vivo* biodistribution of LNA-ASO1 and MOE-ASO1, we performed SplintR PCR to examine tissue concentrations.^18^ In addition to liver, DRG and sciatic nerves, organs such as kidney, heart, lung, spleen, skeletal muscle and skin were also collected for the SplintR analysis from the S331F mice that received repeated ASO treatment. While no tissue concentration was detected in saline control mice, a body-wide biodistribution of ASOs was detected in the ASO-treated groups (Fig. 4D and Supplementary Table 3). In LNA-ASO1-treated mice, kidneys presented the highest concentration of ASOs, at 167.03 × 10^4^ pM, followed by 24.64 × 10^4^ pM in liver. Further assessment of plasma levels of blood urea nitrogen (BUN) excluded potential kidney toxicity in any of the treatment groups (Supplementary Fig. 2). The heart, skin, skeletal muscle and DRG presented similar tissue concentrations in a range between 10^4^ to 10^5^ pM, with a low concentration in the lung and spleen, at 10^3^ pM. In MOE-ASO1-treated mice, high tissue concentrations were detected in a wide range of peripheral organs after 8 weeks of treatment, with liver (5.3 × 10^6^ pM), skin (5.1 × 10^6^ pM) and sciatic nerves (4.9 × 10^6^ pM) presenting the highest tissue concentrations (Fig. 4D and Supplementary Table 3). Our results suggested that repeated subcutaneous injections of LNA-ASO at 15 µg/g or MOE-ASO at 50 µg/g are efficient for delivering ASOs in a wide range of peripheral organs.

### Effective ASO treatment reduces blood levels of 1-deoxySL in S331F mice

**Figure 5:**
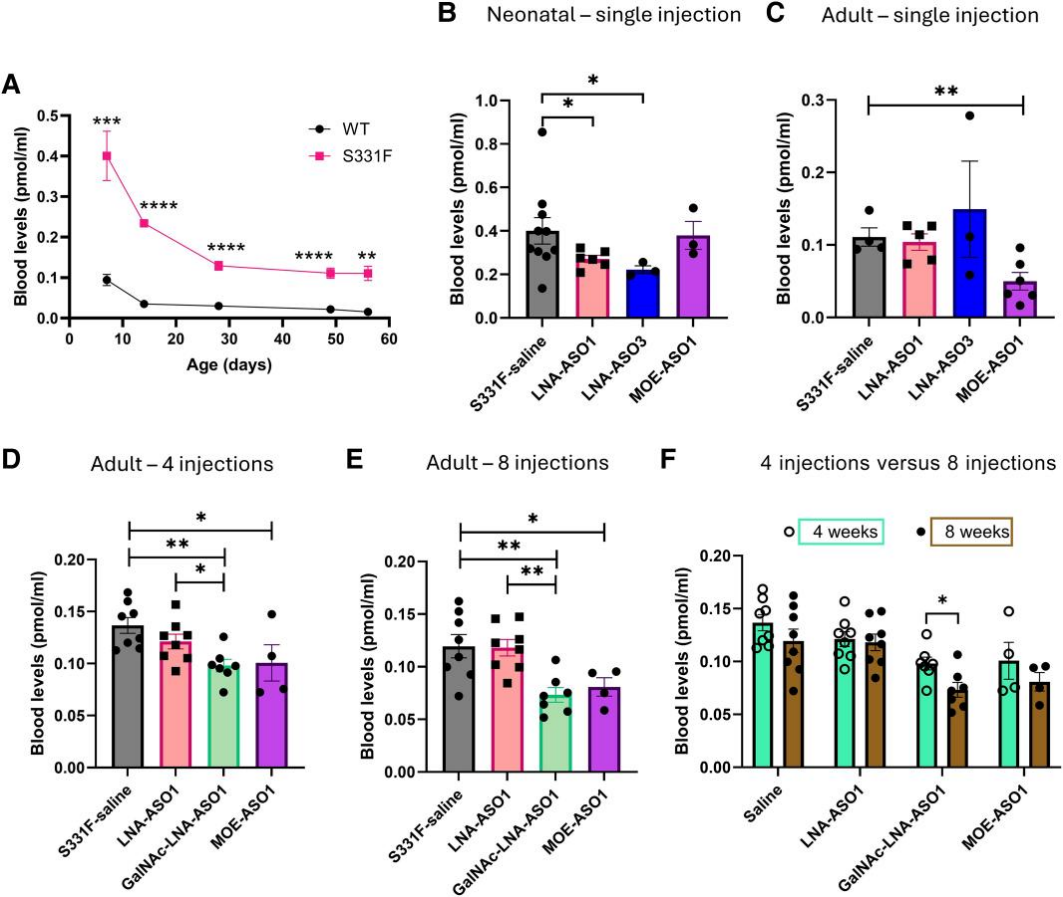
**Plasma levels of 1-deoxySL are increased in S331F mice and reduced after effective ASO treatment**. (**A**) Plasma levels of neurotoxic 1-deoxySL were increased in S331F mice compared to the wild-type (WT), measured at postnatal Days 7, 14, 28 and 56. *n* = 4–13 mice/group/time point. Unpaired Student’s *t*-test was used for data analysis at each time point. (**B** and **C**) 1-deoxySLs were measured in (**B**) neonatal mice and (**C**) adult mice at 7 days after a single subcutaneous injection of ASOs, and (**D** and **E**) in adult mice that received repeated weekly injections of ASOs for 4 weeks (**D**) and 8 weeks (**E**). One-way ANOVA or Kruskal-Wallis, followed by Tukey’s or Dunn’s tests were performed when appropriate. (**F**) Comparison of plasma levels of 1-deoxySL in mice receiving 4 and 8 weeks of treatment. Two-way ANOVA and Sidak’s multiple comparisons test were performed. *n* = 4–10 mice/group. Results are presented as mean ± standard error of the mean. *P < 0.05, **P < 0.01, ***P < 0.001, ****P < 0.0001. ASO = antisense oligonucleotide; 1-deoxySL = 1-deoxysphingolipids; LNA = locked nucleic acid; MOE = 2′-O-methoxy ethyl.

Increased plasma levels of 1-deoxySL, the sum of 1-deoxySO and 1-deoxySA, is a hallmark and biochemical biomarker correlated with clinical measures for HSN1A.^4,21^ To understand the sphingolipid profile and how it changes with disease progression in S331F mice, we conducted a detailed mass spectrometry study on sphingolipids profiling in blood samples collected from mice at different ages, including PND 7, 14, 28, 49 and 56. A distinct sphingolipid signature was detected in the S331F mice compared to age-matched WT controls. The highest levels of 1-deoxySL were detected in the S331F mice at PND7, which then decreased with age (Fig. 5A). At each time point, the plasma levels of 1-deoxySL were consistently and significantly increased in the S331F mice compared to the age-matched WT controls (Fig. 5A). Further studies on gender difference in the plasma levels of 1-deoxySL in 28- and 56-day-old WT and S331F mice showed no difference between the male and female mice in any of the groups (Supplementary Fig. 3).

We continued to measure the plasma levels of 1-deoxySL in response to ASO treatment. Blood samples were collected from S331F mice that received a single or repeated ASO treatment as described earlier. In the single dose group, there were significant reductions in 1-deoxySL from LNA-ASO1- (*P* = 0.021) and LNA-ASO3-treated neonatal mice (*P* = 0.025) compared to saline-treated S331F mice (Fig. 5B). In adult mice, MOE-ASO1 resulted in a significant reduction in 1-deoxySL after a single dose treatment (*P* < 0.001) (Fig. 5C).

To understand the effect of long-term ASO treatment on plasma levels of 1-deoxySL, we collected blood samples from adult S331F mice that received weekly LNA-ASO1, GalNAc-LNA-ASO1 or MOE-ASO1 treatments for 4 or 8 weeks, as described earlier. There was no reduction in 1-deoxySL in LNA-ASO1-treated S331F mice after 4 or 8 weeks of treatment (Fig. 5D and E). Interestingly, the effects were significantly improved by GalNAc conjugation. GalNAc-LNA-ASO1 significantly reduced 1-deoxySL levels after 4 or 8 weeks of treatment, compared to unconjugated LNA-ASO1 (*P* = 0.01 and *P* = 0.002, respectively) or saline (*P* = 0.002 and *P* = 0.005, respectively) (Fig. 5D and E). Eight weeks of GalNAc-LNA-ASO1 treatment also resulted in a significant reduction of 1-deoxySL, further to 4-weeks of treatment (*P* = 0.02) (Fig. 5F). These results suggested that plasma levels of 1-deoxySL can be used as a biochemical biomarker to indicate the therapeutic response, and GalNAc conjugation can improve the therapeutic effect of LNA-ASOs in reducing blood 1-deoxySL levels.

### Transcriptomics studies in S331F mice and the response to ASO treatment

We next investigated the transcriptomic profile in S331F mice to identify underlying molecular pathways and relevant transcripts that may serve as potential molecular markers of the response to efficacious ASO treatment. Next-generation mRNA sequencing was performed on DRG isolated from 3-month-old male WT and S331F mice (*n* = 3/group).

A principal component analysis (PCA) plot showed all WT samples clustered, while the S331F samples were scattered (Supplementary Fig. 4A). Sample correlation showed close correlations among all tested samples (Supplementary Fig. 4B), suggesting no significant segmentation between WT and S331F samples, in line with the subtle phenotype variations between WT and S331F mice at this age.

**Figure 6:**
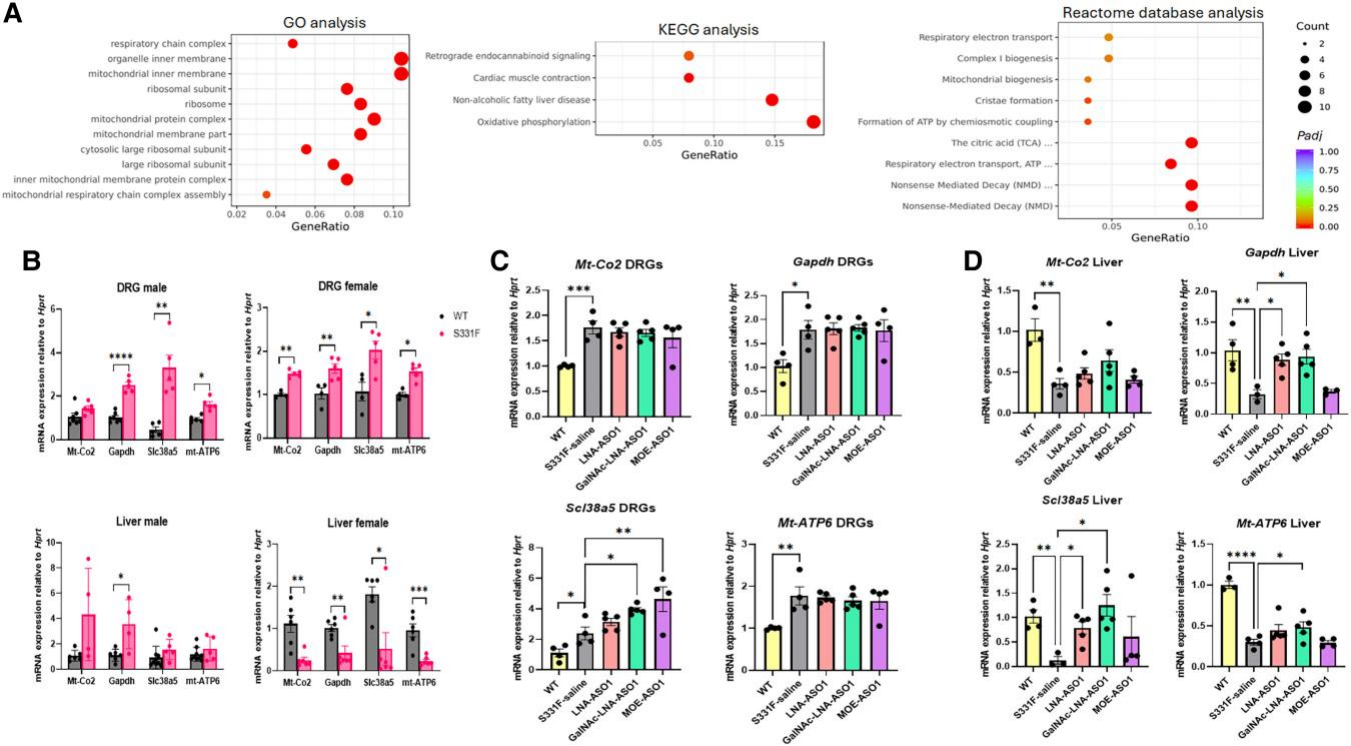
**Transcriptomic studies in S331F mice and the response to ASO treatment**. (**A**) *Left*: The top 11 significantly enriched pathways identified by GO enrichment analysis. *Middle*: The top four significant pathways identified by KEGG enrichment analysis. *Right*: The top nine enriched pathways identified by Reactome database enrichment analysis. (**B**) The expression of four selected DEGs, *Mt-Co2*, *Gapdh*, *Slc385a* and *Mt-ATP6*, in the DRG (*top*), and the liver (*bottom*), from male and female mice, respectively. Two-way ANOVA and Sidak’s multiple comparisons test were performed. *n* = 5–6 mice/group. (**C** and **D**) The expression of *Scl38a5*, *Gapdh*, *Slc385a* and *Mt-ATP6* in DRGs (**C**) and liver (**D**) from WT female mice, S331F female mice treated with saline or different ASOs for 8 weeks. *n* = 4–5 mice/group. One-way ANOVA or Kruskal-Wallis, followed by Tukey’s or Dunn’s tests were performed when appropriate. Data are presented as mean ± standard error of the mean. * *P* < 0.05, ** *P* < 0.01, *** *P* < 0.001, **** *P* < 0.0001. ASO = antisense oligonucleotide; DEG = differentially expressed gene; DRG = dorsal root ganglia; GO = gene ontology; KEGG = Kyoto Encyclopedia of Genes and Genomes; LNA = locked nucleic acid; MOE = 2′-O-methoxy ethyl; WT = wild-type.

Overall, 143 DEGs were identified between the S331F and WT mice (Supplementary Fig. 3C and Supplementary Table 4). GO enrichment analysis of the 143 DEGs identified the 11 most significantly enriched pathways (Fig. 6A, left). KEGG enrichment analysis of the 143 DEGs identified the four most significantly enriched pathways (Fig. 6A, middle), including Oxidative phosphorylation, Non-alcoholic fatty liver disease, Cardiac muscle contraction and Retrograde endocannabinoid signalling. The Reactome database enrichment analysis identified nine enriched pathways (Fig. 6, right), including Nonsense-mediated decay (NMD), Nonsense mediated decay enhanced by the exon junction complex, Respiratory electron transport ATP synthesis by chemiosmotic coupling and heat production by uncoupling proteins, Citric acid cycle respiratory electron transport, Formation of ATP by chemiosmotic coupling, and Cristae formation. These were followed by Mitochondrial biogenesis, Complex I biogenesis and Respiratory electron transport.

From the DEGs identified by the enrichment analysis, pathways associated with mitochondrial function were strongly indicated. We then selected four genes with the highest functional relevance and fold changes, including *Mt-co2*, *Gapdh*, *Mt-ATP6* and *Slc38a5*, for further validation. Their differential expression in liver and DRG were assessed by qRT-PCR in more WT and S331F mice (primers listed in Supplementary Table 1). As the RNA sequencing data were derived from DRG from male mice, to avoid any potential effect of sex difference, the subsequent validation was performed in both male and female mice (*n* = 4 mice/sex/group) (Fig. 6B).

Increased *Mt-Co2*, *Gapdh*, *Slc38a5* and *Mt-ATP6* mRNA expression in DRG from both male and female mice was confirmed by qRT-PCR (Fig. 6B, top). No gender effect on these genes was detected in the DRGs. However, clear discrepancies were detected in the liver, where significantly decreased *Mt-co2*, *Gapdh*, *Slc38a5* and *Mt-ATP6* transcripts were detected in female S331F mice, but significantly increased *Gapdh* transcripts were detected in male S331F mice (Fig. 6B, bottom). Our data suggested a significant gender effect on these genes in the liver; female S331F mice showed more significant changes in the relevant transcriptomics in the liver.

Based on these data, we next investigated the response of these genes to repeated ASO treatment in female S331F mice. qRT-PCRs of *Mt-co2*, *Gapdh*, *Slc38a5* and *Mt-ATP6* transcripts were performed in liver and DRG from female WT (*n* = 4), S331F (*n* = 4) and S331F mice that received 8 weeks of ASO treatment, including LNA-ASO1(*n* = 5), GalNAc-LNA-ASO1 (*n* = 5) and MOE-ASO1 (*n* = 4).

In the DRG, increased transcripts of all four genes were detected in female S331F compared to WT mice. *Mt-Co2*, *Mt-ATP6* and *Gapdh* did not show any response to ASO treatment (Fig. 6C). Interestingly, a significant increase in *Scl38a5* transcripts was detected after treatment with GalNAc-LNA-ASO1 or MOE-ASO1. In liver, compared to saline-treated S331F mice, *Slc38a5*, *Gapdh* and *Mt-ATP6* showed significant responses to GalNAc-LNA-ASO1 treatment, which corrected their expression towards WT levels (Fig. 6D). Mt-Co2 also showed a trend of increased expression after GalNAc-LNA-ASO1 treatment, although not statistically significant (*P* = 0.054). This result was consistent with the earlier data, which showed that GalNAc-LNA-ASO1 had the highest therapeutic effect in the liver compared to the DRG and other unconjugated ASOs (Figs 4 and 5).

## Discussion

SPTLC1-HSN1 is a devastating and progressive neurodegenerative peripheral neuropathy with no disease-modifying treatment currently available. Supplementation with L-serine competes with L-alanine and L-glycine for the binding site of SPT and has been shown to reduce the production of blood 1-deoxySL levels in the C133W-Sptlc1 mouse model and in patients carrying the C133Y mutation.^21^ A pilot clinical study of high doses of L-serine (NCT01733407) suggests it may slow the clinical progression of the disease,^21^ and a subsequent clinical trial is currently ongoing (NCT06113055). At present, there is no other approved therapeutic intervention, and an effective therapy is desperately needed. In this study, we provide necessary *in vivo* evidence as proof-of-concept for the development of allele-specific ASO therapy for SPTLC1-HSN1.

In SPTLC1-HSN1, the primary and most prominent feature is peripheral sensory defects universally present in all patients, although motor defects are also detected in some patients, especially in men. Therefore, in this pilot study, we concentrated only on testing the ASO approach for targeting peripheral organs. In this study, ASOs with MOE or LNA chemical modifications showed efficient biodistribution and target engagement in disease-related peripheral organs, including liver, DRG and sciatic nerves. Moreover, significantly enhanced efficacy was achieved from GalNAc-conjugated LNA-ASO at both mRNA and biochemical levels (Figs 4 and 5). GalNAc-ASO conjugates are used to enhance ASO uptake in hepatocytes.^22,23^ Several US Food and Drug Administration-approved RNA drugs use this approach; for example, eplontersen, an ASO conjugated to GalNAc, and vutrisiran, an siRNA conjugated to GalNAc, are used to treat hereditary transthyretin amyloidosis and have dramatically improved the clinical efficacy and safety profile of treatments compared to their original unconjugated counterparts.^24,25^ The synergistic effect between LNA-ASO and GalNAc conjugation in this study not only suggests liver as one of the key target organs in HSN1, in addition to sensory neurons and peripheral nerves, but also indicates the importance of the conjugation method in enhancing the *in vivo* efficacy of ASOs. While unconjugated gapmer ASOs have demonstrated clinical success, with notable examples including inotersen, volanesorsen and tofersen, we remain cautious about the potential dose-dependent toxicity associated with gapmer ASOs, as evidenced by some setbacks in clinical trials, such as the ASO trials for centronuclear myopathy^26^ and Huntington’s disease.^27^ Therefore, future studies are needed to investigate more formulated ASOs with different conjugates, such as fatty acids or transferrin receptor-binding molecules,^28-30^ beyond GalNAc, to enhance ASO targeting not only in peripheral organs but also in motor neurons within the CNS, maximizing the therapeutic potential of the ASO approach.

The ASOs were in gapmer design to specifically target the transcripts carrying the S331F mutation in the mouse *Sptlc1* gene and exerted allele-specific mRNA silencing via RNase H cleavage. It is noted that the heterozygous S331F mice carry two heterozygous nucleotide mismatches, the synonymous c.990 C>G variant (which was inserted to generate the mutant mouse by CRISPR/Cas9 technology, as it prevents re-cutting of the allele) and the c.992 C>T (p.S331F) missense variant. The former silent variant is not predicted to impact the functional effects of the allele; however, it makes allele discrimination more efficient than for variants with a single nucleotide change, representing the majority of dominant toxic gain-of-function conditions. We have recently reported several strategies to improve the design of allele-specific silencing, including the design of multimers based on the predicted secondary structure of the target sequence and the introduction of additional nucleotide mismatches to increase silencing specificity.^14^ In the real world, the mutation-specific ASO approach presents limited clinical application in conditions where most cases are sporadic. Therefore, ASOs designed to target common mutations or common single nucleotide polymorphisms were developed for allele discrimination. In SPTLC1-HSN1, the p.C133W (c.399T>G) missense mutation represents the most common pathogenic variant, as a founder mutation in cohorts of patients from the UK, Australia, Canada and USA.^1,3,31^ Supported by the present proof-of-concept studies, allele-specific ASOs targeting the founder heterozygous C133W mutation represent a promising experimental ASO therapy for the largest cohort of SPTLC1-HSN1 patients, a project currently under development. In addition to HSN1, successful development of the allele-specific ASO approach may also benefit patients affected by SPTLC1-associated childhood ALS. In contrast to SPTLC1-HSN1, where HSN1-causing variants increase alanine usage by SPT, leading to the formation of deoxy-sphingolipids, ALS-causing variants result in increased production of sphinganine and ceramides.^16^ In addition, SPTLC1-related juvenile ALS patients present early-childhood-onset and exclusive motor involvement, including loss of ambulation and respiratory insufficiency, without any sign of sensory involvement.^16^ This suggests that the allele-specific gene silencing approach can be used in both conditions, with SPTLC1-HSN1 primarily targeting peripheral neuropathy and SPTLC1-ALS targeting motor neurons.

HSN1 has a juvenile to adult onset. While the median age at first symptom onset in patients with HSN1 is 23 years, many patients report sensory symptoms in the early- to mid-teenage years.^32^ In most patients, it is likely that the harm from the accumulated neurotoxic 1-deoxySL begins in childhood. Indeed, in this study, we confirmed the strikingly elevated blood 1-deoxySL levels in neonatal S331F mice (Fig. 5A). Our results also indicated that neonatal mice may have a better response to ASOs than adult mice in abolishing the production of 1-deoxySL (Fig. 5B). While in this study our repeated treatment only focused on young adult mice from 4 weeks old, it is reasonable to assume that early treatment at the presymptomatic stage, such as in children or young teens, may provide more benefit than treatment in adults. To achieve this, a validated clinical biomarker, such as plasma levels of 1-deoxySL, will be needed to guide this therapeutic regimen. Benefiting from advanced genetic diagnosis, many patients carrying the C133W founder mutation in the UK receive presymptomatic diagnoses. These patients are likely to benefit more from a presymptomatic ASO treatment if the hypothesis is confirmed.

The S331F mutation was associated with a severe and early-onset clinical phenotype in two sporadic patients with HSN1A.^33^ In contrast, S331F mice did not exhibit any prominent neurophysiological or pathological phenotype at the current study age, similar to the previously published mouse model of the C133W mutation, where no distinct neuropathy phenotype was detected until adulthood.^34^ This has limited our studies on some relevant clinical outcomes in response to ASO treatment. Increased 1-deoxySL levels were detected in cultured HEK293T cells expressing the p.S331F-SPTLC1 constructs.^35^ In HSN1 patients, plasma levels of 1-deoxySL were reported to be elevated in all patients compared with healthy controls and correlated moderately with the Charcot-Marie-Tooth Neuropathy Score version 2 (CMTNSv2) in males.^32^ Further evidence of plasma 1-deoxySL as a potential biomarker for HSN1 arises from a recent L-serine clinical trial (NCT01733407), where plasma levels of 1-deoxySL were correlated with the CMTNS and reduced after L-serine treatment (59% decrease in serine-treated versus 11% increase in placebo; *P* < 0.001).^36^ In our study, elevated plasma 1-deoxySL levels were detected in S331F mice, starting from neonates (Fig. 5A), and responded to efficient ASO treatment. Therefore, from a drug development perspective, plasma levels of 1-deoxySL in young mice are likely a more efficient biomarker than other late clinical outcomes in aged mice, especially if treatment needs to commence at an early age.

Our transcriptomic studies further indicate mitochondrial dysfunction as a key pathway in the pathogenesis of SPTLC1-HSN1, as supported by the enriched pathways studies and the subsequent validation of the differential expression of *Slc38a5*, *Gapdh*, *Mt-Co2* and *Mt-ATP6* in the DRG and liver of S331F mice and their response to efficient ASO treatment (Fig. 6). *Slc38a5* (Solute Carrier Family 38 Member 5) is a sodium-coupled neutral amino acid transporter, primarily involved in the transport of neutral amino acids including glutamine, alanine and serine.^37^ Deletion of *Slc38a5* in mice leads to the accumulation of 1-deoxySL, mitochondrial abnormalities and motor impairment.^38^ Therefore, the significantly increased expression of *Slc38a5* in the DRG and liver of S331F mice after ASO treatment may be considered a positive therapeutic response (Fig. 6C and D). *Gapdh* (glyceraldehyde-3-phosphate dehydrogenase) is a key enzyme in glycolysis and involved in cell death and mitochondrial function.^39^  *Mt-Co2*, cytochrome c oxidase subunit II, is a crucial component of the cytochrome c oxidase complex (Complex IV) in the mitochondrial respiratory chain involved in the transfer of electrons from cytochrome c to oxygen. *Mt-ATP6* encodes a subunit of ATP synthase (also known as Complex V), a crucial enzyme in the final step of oxidative phosphorylation, which produces ATP, the cell’s main energy source. Both *Mt-Co2* and *Mt-ATP6* are crucial to mitochondrial function. Mitochondrial dysfunction is a hallmark of SPTLC1-HSN1. Mutations in the SPTLC1 protein cause mitochondrial structural abnormalities and endoplasmic reticulum (ER) stress in lymphoblasts.^40^ Exogenous application of 1-deoxySL to cultured primary mouse neurons changes intracellular Ca^2+^ handling in ER and mitochondria and causes an early loss of mitochondrial membrane potential.^5^ Further functional assessment of these pathways, such as the Seahorse assay of patient fibroblasts or induced pluripotent stem cell-derived neurons,^6^ may provide more information on molecular mechanisms and molecular markers for this condition. In addition, a gender difference in mitochondria-related expression of these genes was noticed in the transcriptomic studies of S331F mice, especially in the liver. This is of particular interest, as some males with C133W SPTLC1-HSN1 present earlier and with a more severe phenotype. Our study indicates that further investigation into gender differences in SPTLC1-HSN1, both in patient populations and mouse models, are needed. Gender-specific responses in neurobehavioural studies and treatment efficacy could significantly influence the interpretation of results in mouse models and the design of outcome measures for future ASO therapy trials.

In conclusion, our study shows the therapeutic effect of allele-specific ASOs in selectively silencing mutant transcripts in the S331F mouse model. Our data suggest that targeting the liver with GalNAc-conjugated ASOs is more efficient for reducing plasma 1-deoxySL levels, with enhanced target engagement not only in the liver but also in the DRG and peripheral nerves. Furthermore, we demonstrated the potential of plasma 1-deoxySLs as biochemical biomarkers to assess the therapeutic efficacy of ASO treatment in SPTLC-HSN1 and the need for early therapeutic intervention prior to symptom onset. The identification of mitochondrial pathway involvement provides further insights into the disease pathophysiology and potential molecular markers of SPTLC-HSN1. Our study provides strong *in vivo* evidence for the development of ASO therapy for patients with SPTLC1-HSN1, a rare disease with significant unmet needs and no current therapeutic options.

## Supplementary Material

awaf403_Supplementary_Data

## Data Availability

All data are available in the main text or the Supplementary material. Requests for the S331F mice should be made directly to the MRC Harwell Institute under a material transfer agreement.
