# Supplementary material for: Antisense oligonucleotides reverse SPTLC1-related hereditary sensory neuropathy in a mouse model
Source: Brain. 2025 Oct 22;149(6):2059–71. doi: 10.1093/brain/awaf403 (PMC13233042; doi:10.1093/brain/awaf403)
Supplement: awaf403_Supplementary_Data [file awaf403_supplementary_data.pdf]

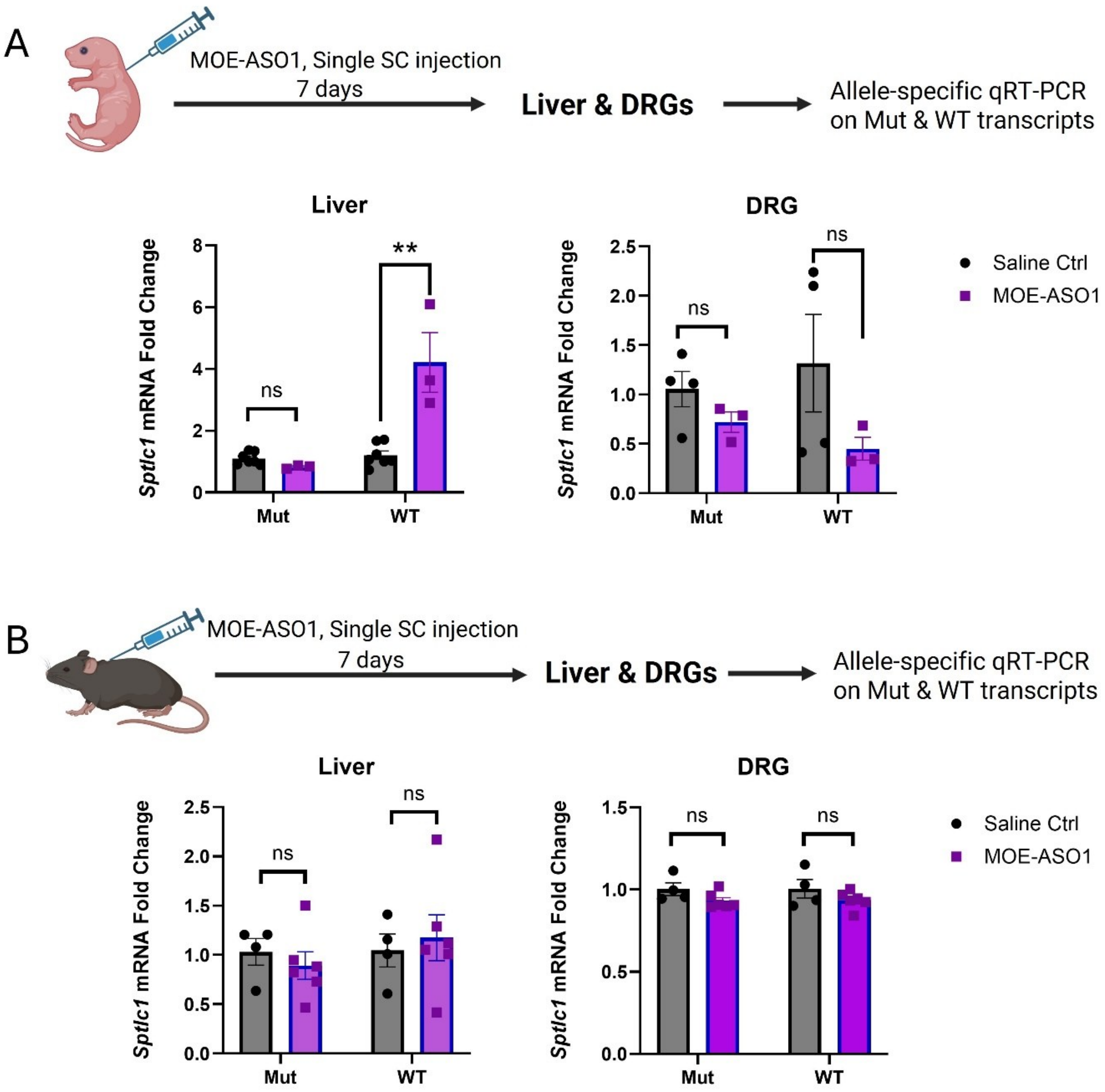

Supplementary Figure 1: Single subcutaneous (SC) injection of MOE-ASO1 at 50 μg/g in (**A**) neonatal at PND3 and (**B**) adult S331F mice at 6 weeks old. liver and DRGs were collected at seven days after the injection. WT and mutant (Mut) *Sptlc1* mRNA levels in ASO-treated mice were measured by allele-specific qRT-PCR and normalized to saline control mice. There were no significant silencing effects on the mutant transcripts in any of the studied groups. Two-way ANOVA and Sidak's multiple comparisons test were performed for statistical significance. N=4-6 mice/group.

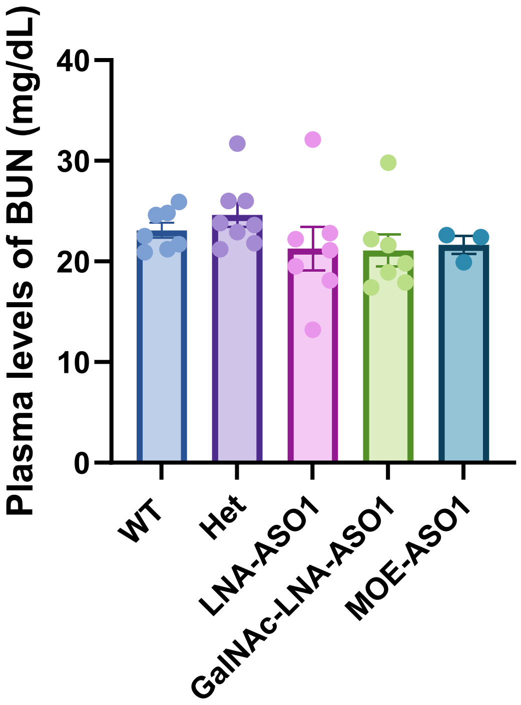

Supplementary Figure 2: The plasma levels of blood urea nitrogen (BUN) were measured using BUN-PIII kit (15809425, DRI-CHEM UK) in mice from the WT and S331F mice after received 8 weeks of ASO treatment. N=3-8 mice/group. One-way ANOVA and Dunnett's multiple comparisons test were performed for statistical significance. Results were presented as Mean ± SEM. No significance (ns) was detected in any of the statistical analysis.

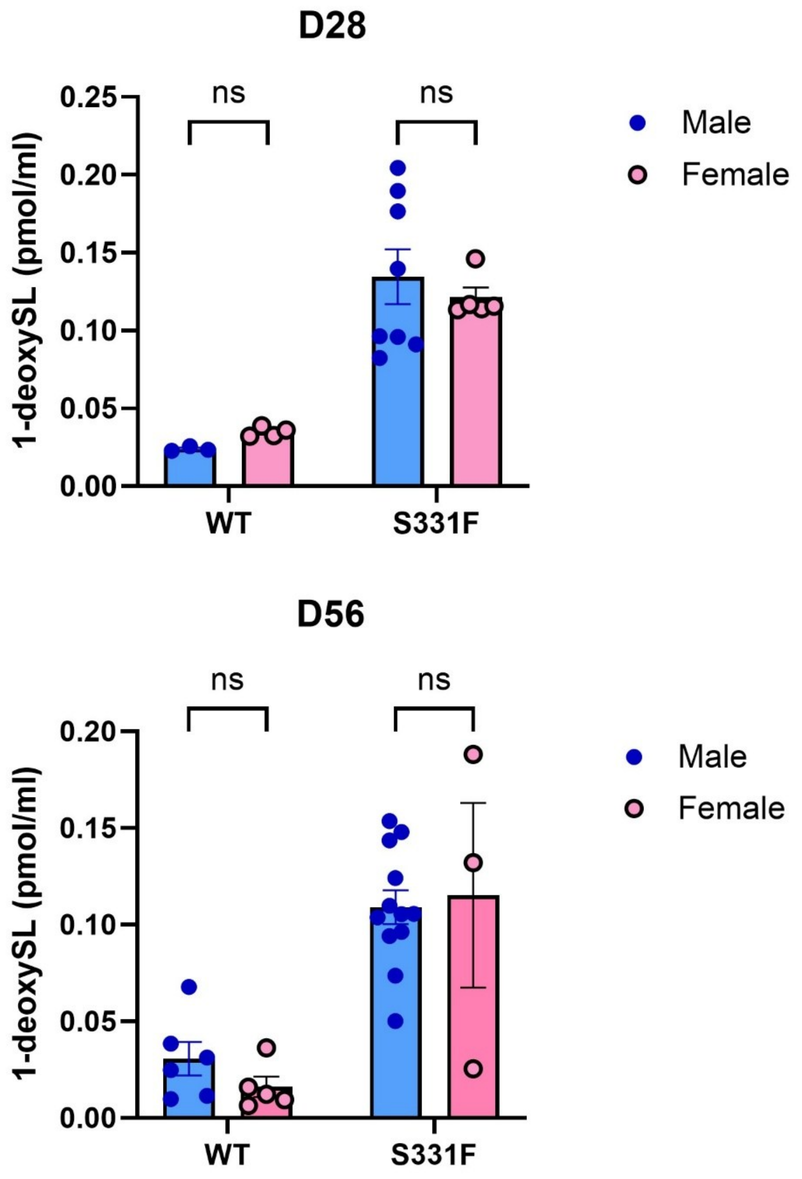

Supplementary Figure 3: The blood levels of 1-deoxySL were measured in male and female mice from the WT and S331F mice at 28 and 56 days of age. N=3-12 mice/sex/group. Two-way ANOVA and Sidak's multiple comparisons test were performed for statistical significance. Results were presented as Mean ± SEM. No significance (ns) was detected in any of the statistical analysis.

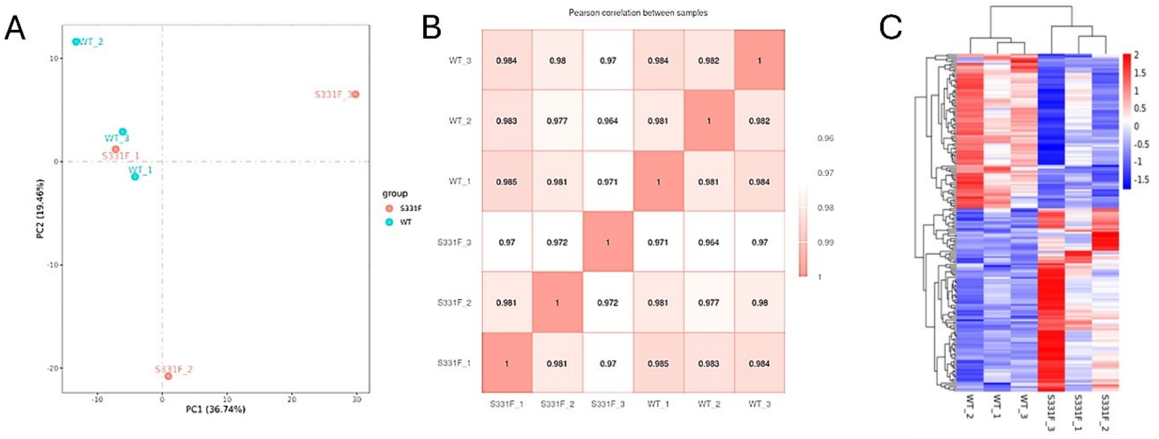

Supplementary Figure 4: RNA sequencing result showing the correlation (**A, B**) between the WT and S331F mice. (**C**) Heatmap of the DEGs identified in S331F mice.

Supplementary Table 1

Primer sequences for qRT-PCR

Gene	Primer sequence	Product Size (bp)
**WT *Sptlc1***	F: TGGTTGACCATCAGCGGCTCCC<br>R: TGGATTCTCTTCCATGATGTTG	110
**Mutant *Sptlc1***	F: TGACCATCAGCGGCTGTT<br>R: TGGATTCTCTTCCATGATGTTG	106
Hprt1	F: CCCTGGTTAAGCAGTACAGC<br>R: ACAAAGTCTGGCCTGTATCCA	94
Mt-ATP6	F: CCTTCCACAAGGAACTCCAA<br>R: TGTAAGCCGGACTGCTAATG	100
Mt-Co2	F: CATCCCAGGCCGACTAAAT<br>R: TGGGCATAAAGCTATGGTTAGA	104
Slc38a5	F: TCAACCTCAGCAACGCTATC<br>R: GCAGGAGGTGAATGGAGTAAG	135
Gapdh	F: AACAGCAACTCCCACTCTTC<br>R: CCTGTTGCTGTAGCCGTATT	111

Supplementary Table 2

IC50 of ASOs on downregulating the mRNA expression of the mutant and WT *Sptlc1* transcripts

ASO	IC50 on Mut transcripts	IC50 on WT transcripts	IC50 WT/Mut
**Fibroblasts treated with ASOs (nM) under Lipofectamine 2000 transfection**			
MOE-ASO1	0.67	13.56	20.24
MOE-ASO2	0.62	7.31	11.81
MOE-ASO3	0.12	0.39	3.25
LNA-ASO1	0.08	3.00	37.50
LNA-ASO2	0.60	10.57	17.67
LNA-ASO3	0.24	1.61	6.72
**Fibroblasts treated with ASOs (µM) under gymnosis**			
MOE-ASO1	0.81	35.95	44.38
MOE-ASO2	4.678	3518	752
MOE-ASO3	8.37	13.3	1.59
LNA-ASO1	5.27	11.63	2.21
LNA-ASO2	6.61	12.88	1.95
LNA-ASO3	0.99	6.63	6.7

Supplementary Table 3

Tissue concentrations of ASOs in Sptlc1 mice after repeated weekly injections for 8 weeks

Tissue	LNA-ASO1 (fM)	MOE-ASO1 (fM)	Saline Ctrl (fM)
Kidneys	167.03 ± 62.34 ($\times 10^4$)	311.29 ± 74.09 ($\times 10^4$)	2.37 ± 0.17
Liver	24.64 ± 18.99 ($\times 10^4$)	531.21 ± 97.19 ($\times 10^4$)	0.66 ± 0.12
DRGs	2.96 ± 0.27 ($\times 10^4$)	160.32 ± 24.61 ($\times 10^4$)	5.71 ± 0.19
Sciatic nerves	4.48 ± 1.46 ($\times 10^4$)	494.45 ± 10.41 ($\times 10^4$)	5.71 ± 0.19
Heart	5.10 ± 1.97 ($\times 10^4$)	263.47 ± 56.69 ($\times 10^4$)	0.52 ± 0.07
Lung	0.80 ± 0.45 ($\times 10^4$)	282.80 ± 57.23 ($\times 10^4$)	0.74 ± 0.15
Skeletal muscle	3.13 ± 0.99 ($\times 10^4$)	159.84 ± 21.47 ($\times 10^4$)	5.24 ± 0.27
Skin	3.97 ± 2.32 ($\times 10^4$)	513.16 ± 78.74 ($\times 10^4$)	3.81 ± 0.22
Spleen	0.52 ± 0.19 ($\times 10^4$)	244.19 ± 30.27 ($\times 10^4$)	0.65 ± 0.08

fM: femtomolar (10^{-15} mol/L).

Supplementary Table 4: List of DEGs between WT and S331F mice

Supplementary Table 3: List of DEGs between WT and S331F mice									
gene_ID	Gene fpkm						log2FoldChange	pvalue	gene_name
	S331F_1	S331F_2	S331F_3	WT_1	WT_2	WT_3			
ENSMUSG00000022066	17.6595	3.97581	23.9411	164.821	137.526	129.767	-3.26352	1.25E-16	Entpd4b
ENSMUSG00000064354	1015.84	638.117	1183.88	499.369	454.31	465.084	0.999383	1.25E-07	mt-Co2
ENSMUSG00002076161	234.62	528.782	263.352	775.053	667.716	557.478	-0.96233	6.47E-05	Rn7sk
ENSMUSG00000038541	183.323	84.4859	35.9117	35.3188	25.6084	33.2203	1.699111	0.00022	Srd5a2
ENSMUSG00000019841	391.874	403.544	275.323	571.969	585.200	504.534	-0.63061	0.00025	Rev3l
ENSMUSG00000031592	1101.62	793.174	663.169	1142.95	1601.95	1266.52	-0.6471	0.00029	Pcm1
ENSMUSG00000092274	8233.56	7937.70	6863.92	5519.55	5722.06	6292.14	0.393984	0.00035	Neat1
ENSMUSG00000071753	762.725	751.428	653.593	990.891	1035.71	946.779	-0.45454	0.00036	Cdr1os
ENSMUSG00000086290	180.800	201.772	258.564	126.559	128.042	116.271	0.784785	0.00037	Snhg12
ENSMUSG00000035842	77.3657	77.5283	108.932	33.3567	33.1961	49.8305	1.178794	0.00051	Ddx11
ENSMUSG00000047910	50.4559	26.8367	21.5470	65.7323	100.536	84.0889	-1.32228	0.00056	Pcdhb16
ENSMUSG00000024186	1190.76	1381.59	1408.93	1070.35	867.841	985.190	0.444922	0.00068	Rgs11
ENSMUSG00000039899	429.716	335.956	351.934	586.686	596.581	468.199	-0.56161	0.00069	Fgl2
ENSMUSG00000021268	39475.0	46184.0	39872.7	36227.3	30163.9	29530.8	0.388115	0.00073	Meg3
ENSMUSG00000021647	384.305	354.841	575.784	282.551	262.723	287.563	0.655761	0.00079	Cartpt
ENSMUSG00000055540	110.162	89.4557	58.6558	142.256	214.352	141.186	-0.93787	0.00093	Epha6
ENSMUSG00000072849	41.2056	132.195	32.3205	13.7351	27.5053	21.8008	1.707169	0.00102	Serpina1e
ENSMUSG00000024383	480.172	361.798	356.723	518.991	704.706	559.555	-0.57057	0.00108	Map3k2
ENSMUSG00000045573	243.870	127.226	129.282	103.994	76.8253	77.8601	0.957302	0.00114	Penk
ENSMUSG00000040651	264.893	205.748	257.367	331.605	436.291	337.394	-0.60279	0.00124	Tasor
ENSMUSG00000004263	403.647	373.726	494.384	324.737	252.290	278.220	0.570485	0.00125	Atn1
ENSMUSG00000001056	576.879	709.682	910.960	484.653	475.178	541.906	0.546919	0.00132	Nhp2
ENSMUSG00000079523	7411.97	7945.65	13045.5	6784.17	5917.44	6766.56	0.544682	0.00133	Tmsb10
ENSMUSG00000057789	223.687	270.355	231.032	147.162	133.733	178.559	0.659357	0.00134	Bak1
ENSMUSG00000042312	1437.99	1472.04	2330.67	1266.57	1120.13	1219.80	0.53792	0.00134	S100a13
ENSMUSG00000030660	291.803	191.832	129.282	326.699	425.858	310.402	-0.78751	0.00146	Pik3c2a
ENSMUSG00000003452	1044.43	860.763	906.172	1152.76	1435.97	1205.27	-0.43172	0.00147	Bicd1
ENSMUSG00000075316	5869.70	4604.98	4654.15	5754.03	7631.31	6903.60	-0.42311	0.00153	Scn9a
ENSMUSG00000063663	103.434	85.4799	86.1881	157.953	189.692	127.690	-0.78669	0.00169	Brwd3
ENSMUSG00000025321	1617.11	1222.56	1064.18	1644.29	2320.88	1667.24	-0.52771	0.00176	Itgb8
ENSMUSG00000003226	1772.68	1400.48	1025.87	1717.87	2388.22	2021.25	-0.5438	0.00186	Ranbp2
ENSMUSG00000030235	47.0921	38.7641	29.9264	92.2215	106.227	53.9830	-1.11684	0.00212	Slco1c1
ENSMUSG00000038665	1337.92	1096.33	697.884	1286.19	1834.32	1599.76	-0.58989	0.00218	Dgki
ENSMUSG00000060636	1535.54	1560.50	2548.53	1415.69	1119.18	1343.34	0.540433	0.00221	Rpl35a
ENSMUSG00000025907	1870.23	1681.76	1388.58	1930.76	2521.95	2194.61	-0.4271	0.00235	Rb1cc1
ENSMUSG00000022762	224.528	153.068	131.676	245.270	289.280	281.334	-0.67259	0.00236	Ncam2
ENSMUSG00000064349	35.3191	92.4376	69.4293	39.2432	19.9176	12.4576	1.454697	0.00237	mt-Tc
ENSMUSG00000057666	846.818	169.965	5325.70	200.140	134.681	174.406	3.638586	0.00239	Gapdh
ENSMUSG00000038174	1034.34	825.974	823.575	1071.34	1394.23	1179.32	-0.44031	0.00240	Fam126b
ENSMUSG00000073664	951.094	726.579	673.943	950.666	1340.17	1042.28	-0.50157	0.00244	Nbeal1
ENSMUSG00000080921	182.482	247.494	477.625	148.143	172.619	181.673	0.84817	0.00246	Rpl38-ps2
ENSMUSG00000050552	941.002	1021.78	1525.05	852.558	775.840	820.127	0.508579	0.00248	Lamtor4

ENSMUSG00000062328	560.060	616.250	773.298	462.088	412.580	512.839	0.488852	0.00259	Rpl17
ENSMUSG00000037369	335.531	290.234	275.323	378.697	431.549	467.161	-0.50046	0.00262	Kdm6a
ENSMUSG00000091086	328.804	335.956	547.055	284.513	253.239	246.038	0.624575	0.00263	Rpl6l
ENSMUSG00000037857	933.434	835.914	842.728	1057.60	1287.06	1083.81	-0.39183	0.00265	Nufip2
ENSMUSG00000024294	2472.34	2073.38	1939.23	2667.55	2985.75	2700.19	-0.36447	0.00281	Mib1
ENSMUSG00000007653	97.5481	59.6371	17.9558	98.1080	184.001	130.805	-1.22217	0.00289	Gabrb2
ENSMUSG00000056629	1326.99	1445.20	2157.09	1288.15	1004.42	1179.32	0.504453	0.00304	Fkbp2
ENSMUSG00000075470	681.154	546.674	505.158	700.491	887.759	798.326	-0.45932	0.00316	Alg10b
ENSMUSG00000041841	1901.34	1771.22	3284.72	1740.43	1374.32	1618.45	0.554864	0.00324	Rpl37
ENSMUSG00000035215	516.332	645.075	997.148	466.994	446.725	518.029	0.589698	0.00333	Lsm7
ENSMUSG00000022354	867.000	931.333	1610.04	841.766	697.118	751.610	0.571591	0.00341	Ndufb9
ENSMUSG00000086841	247.234	317.070	463.261	226.629	193.486	232.542	0.650693	0.00347	2410006H16Rik
ENSMUSG00000001025	7895.51	8416.79	13814.0	7910.45	6421.07	6988.73	0.49856	0.00358	S100a6
ENSMUSG00000006740	10123.1	8352.18	8450.02	10274.8	12773.8	11224.3	-0.34797	0.00359	Kif5b
ENSMUSG00000050071	150.526	191.832	253.776	130.483	108.124	126.652	0.70167	0.00368	Bex1
ENSMUSG00000044098	165.663	88.4618	49.0793	199.159	227.630	161.949	-0.94583	0.00375	Rsbn1
ENSMUSG00000024309	704.701	773.295	1065.38	618.080	515.014	669.597	0.49489	0.00377	Pfdn6
ENSMUSG00000056342	1745.77	1250.39	910.960	1701.19	2242.16	1750.29	-0.54171	0.00382	Usp34
ENSMUSG00000074884	1086.48	1149.00	1520.26	945.761	935.182	936.398	0.413095	0.00387	Serf2
ENSMUSG00000026384	212.755	239.542	198.711	285.494	361.363	294.830	-0.53193	0.00393	Ptpn4
ENSMUSG00000043467	324.599	173.941	129.282	344.359	435.343	297.944	-0.77289	0.00393	Zbtb37
ENSMUSG00000080893	415.420	411.496	599.725	183.462	283.589	413.178	0.695843	0.00398	Ndufa12-ps
ENSMUSG00000060803	1242.89	1439.24	2103.22	1234.19	1062.27	1120.14	0.484629	0.00407	Gstp1
ENSMUSG00000039701	218.642	209.724	186.740	284.513	345.239	264.724	-0.53846	0.00411	Usp53
ENSMUSG00000059534	1577.58	1771.22	2878.92	1580.52	1328.79	1434.70	0.518571	0.00415	Uqcr10
ENSMUSG00000083596	627.335	626.190	1070.16	585.704	475.178	514.915	0.557963	0.00421	Rpl21-ps15
ENSMUSG00000053964	171.550	166.984	233.426	134.408	110.021	118.347	0.652838	0.00445	Lgals4
ENSMUSG00000024726	177.436	176.923	148.435	251.156	258.929	226.313	-0.54775	0.00465	Carnmt1
ENSMUSG00000024846	337.213	281.288	502.764	224.667	259.878	255.381	0.596256	0.00489	Cst6
ENSMUSG00000079480	285.075	407.520	651.199	252.137	272.208	316.631	0.672599	0.00492	Pin4
ENSMUSG00000054428	1632.24	1958.08	3096.78	1652.13	1496.67	1548.89	0.508335	0.00492	Atpif1
ENSMUSG00000046449	42.8875	41.7460	35.9117	50.0350	106.227	88.2415	-1.01692	0.00494	Nexmif
ENSMUSG00000015243	1406.87	1378.61	1028.27	1439.24	1920.63	1752.37	-0.42152	0.00494	Abca1
ENSMUSG00000040455	1202.53	1093.34	875.048	1235.18	1535.55	1429.51	-0.40393	0.00503	Usp45
ENSMUSG00000042473	131.185	86.4739	83.794	139.313	184.001	175.444	-0.71865	0.00508	Tbc1d8b
ENSMUSG00000040584	519.695	431.375	323.205	590.610	680.046	539.830	-0.50312	0.00514	Abcb1a
ENSMUSG00000024529	648.358	417.460	454.881	738.753	830.851	600.042	-0.51065	0.00522	Lox
ENSMUSG00000032517	222.847	205.748	264.549	110.862	184.001	150.529	0.635503	0.00527	Mobp
ENSMUSG00000061518	2849.07	3156.79	4844.49	2865.73	2113.17	2760.40	0.486935	0.00530	Cox5b
ENSMUSG00000023845	5326.46	4041.41	3631.87	5061.39	6433.40	5681.71	-0.40148	0.00530	Lnpep
ENSMUSG00000058838	43.7284	59.6371	144.843	46.1107	24.6599	34.2584	1.231691	0.00536	Rps27a-ps2
ENSMUSG00000020326	1679.34	1525.71	1596.87	1845.41	2147.31	1982.83	-0.31527	0.00546	Ccng1
ENSMUSG00000064357	383.465	1877.57	586.558	273.721	285.486	191.017	1.924229	0.00568	mt-Atp6
ENSMUSG00000033938	1420.33	1437.25	2367.77	1303.85	1122.02	1311.16	0.482469	0.00582	Ndufb7
ENSMUSG00000059540	827.477	931.333	1065.38	766.223	697.118	712.161	0.37477	0.00617	Tcea2
ENSMUSG00000022324	2064.48	1830.86	1995.49	2468.39	2535.23	2257.94	-0.3018	0.00622	Matn2

ENSMUSG00000025938	740.861	632.154	434.531	682.831	1095.47	855.423	-0.5407	0.00628	Slco5a1
ENSMUSG00000048758	1682.70	1653.93	2914.83	1541.27	1414.15	1489.72	0.490888	0.00628	Rpl29
ENSMUSG00000034566	2971.85	3032.55	4783.44	2883.39	2376.84	2655.55	0.445983	0.00634	Atp5h
ENSMUSG00000007613	329.645	282.282	256.170	366.924	440.085	393.453	-0.46446	0.00638	Tgfbr1
ENSMUSG00000054312	715.633	843.865	1327.53	706.377	635.468	676.864	0.513941	0.00659	Mrps21
ENSMUSG00000050945	51.2968	51.6855	43.0940	81.4296	105.279	76.8220	-0.84834	0.00660	Zfp438
ENSMUSG00000020875	119.412	95.4194	74.2175	135.389	171.671	161.949	-0.68993	0.00662	Hoxb9
ENSMUSG00000032253	453.262	404.538	386.649	558.234	644.953	478.580	-0.43337	0.00665	Phip
ENSMUSG00000019577	132.867	126.232	100.552	171.689	215.300	167.139	-0.61942	0.00666	Pdk4
ENSMUSG00000048960	578.561	375.714	368.693	574.913	768.253	563.707	-0.52457	0.00668	Prex2
ENSMUSG00000063316	2764.14	3148.84	4648.17	2946.18	2112.22	2583.92	0.466113	0.00677	Rpl27
ENSMUSG00000067714	196.778	253.458	175.967	277.645	302.559	320.789	-0.52284	0.00685	Lpar5
ENSMUSG00000022579	67.2745	40.7520	61.0499	31.3945	18.9692	28.0296	1.111776	0.00692	Gpihbp1
ENSMUSG00000045282	155.572	193.820	228.637	133.426	129.939	113.156	0.613056	0.00703	Tmem86b
ENSMUSG00000014313	2925.60	3367.51	5097.06	2967.76	2696.47	2701.22	0.44459	0.00710	Cox6c
ENSMUSG00000035284	1419.49	1113.22	925.325	1375.47	1814.40	1479.34	-0.43169	0.00724	Vps13c
ENSMUSG00000079277	58.0243	49.6976	67.0352	27.4702	26.5569	32.1822	1.018182	0.00727	Hoxd3
ENSMUSG00000059742	2184.74	1488.94	1365.84	2074.00	2631.97	2140.63	-0.44097	0.00741	Kcnh7
ENSMUSG00000036745	17265.1	14367.5	13664.4	16797.0	20311.2	19447.4	-0.32013	0.00755	Ttll7
ENSMUSG00000034218	328.804	215.687	131.676	299.229	450.518	350.889	-0.6966	0.00757	Atm
ENSMUSG00000081094	159.777	112.316	149.632	102.032	73.9799	84.0889	0.698203	0.00759	Rpl19-ps11
ENSMUSG00000015981	1043.59	1112.23	1231.77	965.383	830.851	878.262	0.33996	0.00771	Stk32c
ENSMUSG00000025650	274.984	414.478	593.740	316.888	238.063	277.182	0.621167	0.00776	Col7a1
ENSMUSG00000010406	576.879	559.595	913.354	522.915	418.271	503.495	0.502473	0.00788	Mrpl52
ENSMUSG00000073616	1218.51	1297.10	2052.95	1196.91	1046.15	1076.54	0.459244	0.008	Cops9
ENSMUSG00000033960	611.357	599.353	526.705	676.945	813.779	756.800	-0.36994	0.00804	Jcad
ENSMUSG00000092083	2669.95	2324.85	1954.79	2737.21	3350.9	2820.61	-0.35751	0.0081	Kcnb2
ENSMUSG00000053332	999.868	989.977	1571.73	966.364	778.686	839.851	0.460775	0.00815	Gas5
ENSMUSG00000040760	1570.86	1465.08	1230.57	1671.76	2035.39	1730.5	-0.34893	0.00826	Appl1
ENSMUSG00000071669	294.326	263.397	146.041	299.229	411.631	369.576	-0.61264	0.00829	Snx29
ENSMUSG00000003955	625.653	622.214	989.966	557.253	538.725	514.915	0.471575	0.00836	Fam162a
ENSMUSG00000024597	3489.86	2700.57	2769.99	3623.12	4072.69	3569.11	-0.32969	0.00842	Slc12a2
ENSMUSG00000038349	284.235	257.433	201.105	307.078	408.786	340.508	-0.50418	0.00843	Plcl1
ENSMUSG00000038489	710.587	820.011	1339.50	676.945	680.994	660.254	0.5057	0.00846	Polr2l
ENSMUSG00000019966	1072.18	925.370	966.025	1126.28	1457.78	1210.46	-0.35615	0.00849	Kitl
ENSMUSG00000033256	639.108	629.172	727.810	556.272	514.065	468.199	0.374046	0.00852	Shf
ENSMUSG00000074505	743.383	797.150	538.675	813.315	1008.21	970.656	-0.42332	0.00854	Fat3
ENSMUSG00000028648	2087.19	2149.92	3224.87	1954.31	1745.16	1920.55	0.408088	0.00855	Ndufs5
ENSMUSG00000035674	1312.69	1264.30	2080.48	1255.78	1058.48	1083.81	0.453481	0.00856	Ndufa3
ENSMUSG00000031167	1879.48	1690.71	2018.23	1525.58	1298.44	1614.30	0.33234	0.00857	Rbm3
ENSMUSG00000037705	161.458	154.062	122.099	212.894	212.455	213.856	-0.54152	0.00857	Tecta
ENSMUSG00000056071	22.7051	8.94557	25.1382	42.1864	43.6291	48.7923	-1.24909	0.00859	S100a9
ENSMUSG00000089774	835.886	864.739	854.698	1004.62	1270.93	991.419	-0.35496	0.00871	Slc5a3
ENSMUSG00000031170	1.68186	0	0	11.7729	12.3299	4.15254	-3.91152	0.00871	Slc38a5*
ENSMUSG00000036202	246.393	208.730	179.558	318.851	369.899	239.809	-0.546	0.00881	Rif1
ENSMUSG00000038690	2573.25	2842.70	4322.57	2433.07	2248.8	2561.08	0.426414	0.00886	Atp5j2

ENSMUSG00000058546	1268.96	1178.82	1721.36	1195.93	1006.31	930.169	0.411145	0.00892	Rpl23a
ENSMUSG00000022141	709.746	592.395	499.172	743.658	878.274	767.182	-0.40479	0.00902	Nipbl
ENSMUSG00000038039	1067.14	869.708	796.043	1051.71	1438.81	1128.45	-0.40382	0.00912	Gcc2
ENSMUSG00000058809	85.7750	84.4859	52.6705	309.040	75.8768	329.089	-1.67728	0.00924	Hspd1-ps3
ENSMUSG00000035954	681.995	588.420	474.034	627.891	901.986	857.500	-0.45025	0.00946	Dock4
ENSMUSG00000027177	1799.59	1544.60	1398.16	1896.42	2052.46	1971.42	-0.31887	0.00971	Hipk3
ENSMUSG00000029826	434.761	410.502	387.846	502.313	540.622	553.326	-0.37073	0.00996	Zc3hav1
ENSMUSG00000022217	1205.05	1497.88	1806.35	1229.29	983.553	1205.27	0.398557	0.00998	Emc9

*Slc38a5 gene has low FKPM (<10). However, considering its involvement in serine metabolism and mitochondrial function as previously reported (33,34), we have included Slc38a5 on the list and performed the subsequent validation in the DRGs and liver.

Supplementary Table 5

Primers and probes used in SplintR PCR

Primer/Probe	Sequence	Note
**Probe A**	CTCGACCTCTCTATGGGCAGTCACG ACAGAGCGGCTG	
**Probe B**	pTTCGGTCAACGCTGAGTCGGAGAC ACGCAGGGCTTAA	p: 5’ phosphate
**Primer F**	GCTCGACCTCTCTATGGGC	
**Primer R**	TTAAGCCCTGCGTGTCTCC	qPCR Primer & Probe Set
**Probe**	ACCGAACAGCCGCTCTGTCGTG	
